# Sterols, Oxysterols, and Accessible Cholesterol: Signalling for Homeostasis, in Immunity and During Development

**DOI:** 10.3389/fphys.2021.723224

**Published:** 2021-10-08

**Authors:** William J. Griffiths, Yuqin Wang

**Affiliations:** Swansea University Medical School, Swansea, United Kingdom

**Keywords:** 25-hydroxycholesterol, SREBP pathway, INSIG, HMG-CoA reductase, SARS-CoV-2, hedgehog signalling pathway, cholesterol dependent cytolysin

## Abstract

In this article we discuss the concept of accessible plasma membrane cholesterol and its involvement as a signalling molecule. Changes in plasma membrane accessible cholesterol, although only being minor in the context of total cholesterol plasma membrane cholesterol and total cell cholesterol, are a key regulator of overall cellular cholesterol homeostasis by the SREBP pathway. Accessible cholesterol also provides the second messenger between patched 1 and smoothened in the hedgehog signalling pathway important during development, and its depletion may provide a mechanism of resistance to microbial pathogens including SARS-CoV-2. We revise the hypothesis that oxysterols are a signalling form of cholesterol, in this instance as a rapidly acting and paracrine version of accessible cholesterol.

## Introduction

Cholesterol is the dominant sterol in animal cells. It is present at a level of about 10–20 fmol/cell (Radhakrishnan et al., 2008; Infante and Radhakrishnan, 2017) and can be found in the membrane of every organelle, with about 60–90% of cellular cholesterol present in the plasma membrane (Lange et al., 1989), where its concentration may be 45 mole % of total lipids (Lange et al., 1989; Das et al., 2013, 2014). The unit mole % of total lipids is used to convey the % contribution in moles of a defined lipid, i.e., cholesterol, to the total lipid content of a membrane or cell. Intracellularly, cholesterol is trafficked between organelles *via* vesicular transport or non-vesicular transport mechanisms involving membrane contact sites (Sandhu et al., 2018; Hoglinger et al., 2019; Naito et al., 2019; Ferrari et al., 2020; Trinh et al., 2020). It may be imported to cells *via* lipoprotein up-take or it can be synthesised *de novo*. Almost every vertebrate cell has the machinery to synthesise cholesterol (Nes, 2011; Cerqueira et al., 2016), and most if not all have the capacity to metabolise it, the first step of which is oxidation to an oxysterol (Schroepfer, 2000). Many invertebrates, including insects and nematodes, are sterol auxotrophs, relying on sterols consumption as part of the diet (Carvalho et al., 2010). Oxysterols are oxidised forms of cholesterol and can cross membranes far quicker than cholesterol (Lange et al., 1995; Meaney et al., 2002). Different cells and tissues generate different oxysterols and like cholesterol can be esterified and transported in the circulation with lipoproteins (Dzeletovic et al., 1995). A small fraction of cholesterol is converted to steroid hormones, but the majority is converted to bile acids (Russell, 2003; Griffiths and Wang, 2020). Bile acid biosynthesis proceeds predominantly in the liver but minor pathways may be initiated extrahepatically (Babiker et al., 1999; Meaney et al., 2007; Ogundare et al., 2010; Griffiths and Wang, 2020). Bile acids have many functions, they provide an elimination form of cholesterol; in the biliary tract they solubilise and transport cholesterol; and in the small intestine they solubilise dietary lipids and act as antimicrobials. Remarkably, 90% of bile acids entering the small intestine are reabsorbed, and recycled to the liver *via* the enterohepatic system (Hofmann and Hagey, 2014). While oxysterols and bile acids are *bona fide* signalling molecules (Evans and Mangelsdorf, 2014), so is cholesterol in its “accessible” form (Radhakrishnan et al., 2020).

## Cholesterol Biosynthesis, Uptake, Genes, Enzymes, and Regulation

### Cholesterol Biosynthesis

**Figure 1:**
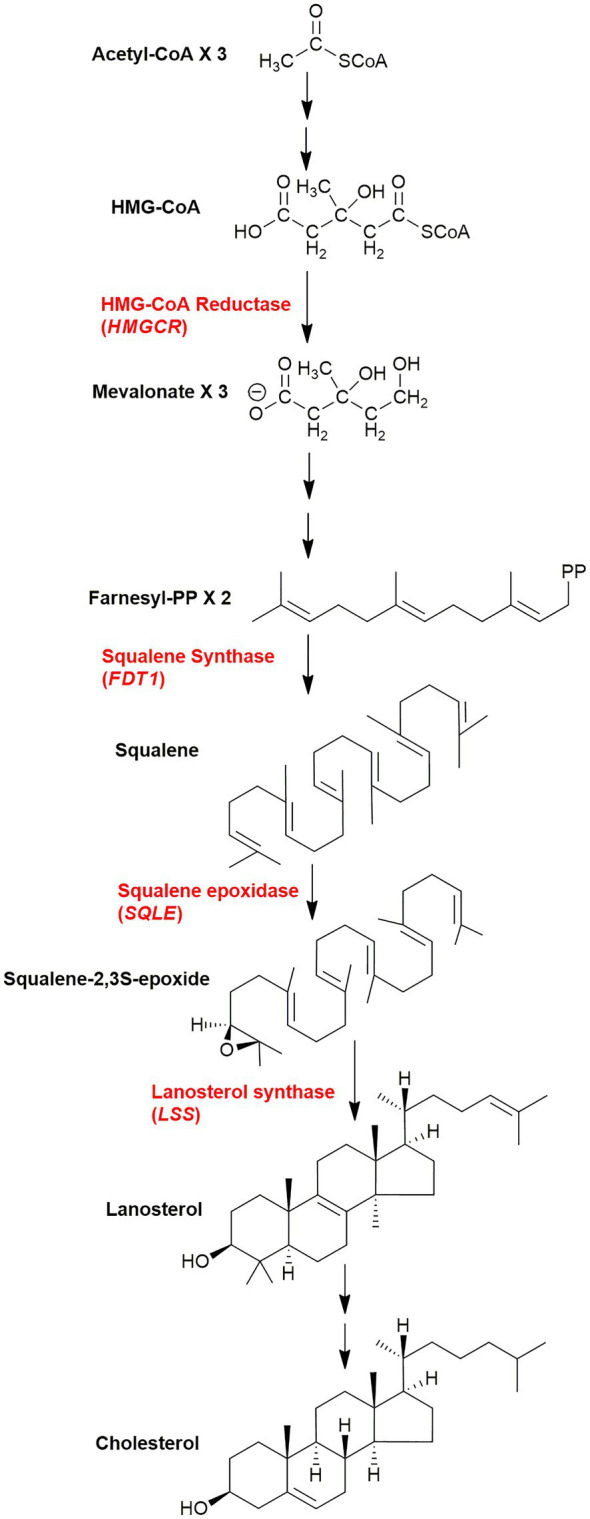
Simplified scheme of the cholesterol biosynthetic pathway. Key enzymes are in red, genes in parenthesis. The pathway post squalene can be found in detail in Figure 6. The entire pathway including all steps and enzymes can be found in Mazein et al. (2013).

Cholesterol is synthesised from acetyl-CoA in a pathway involving at least 20 enzymes (Figure 1; Goldstein et al., 2006; Nes, 2011; Mazein et al., 2013; Cerqueira et al., 2016). There are five key intermediates in this pathway (i) 6-carbon mevalonate generated by reduction of 3-hydroxy-3-methylglutaryl-CoA (HMG-CoA) by HMG-CoA reductase, this represents the rate determining step of the pathway, (ii) 15-carbon farnesyl pyrophosphate (farnesyl-PP), this provides a branch point to nonsteroidal isoprenoids, e.g., geranylgeraniol, dolichols, and ubiquinone, (iii) 30-carbon squalene generated from two farnesyl-PP substrates, the precursor of (iv) squalene-2,3S-epoxide, and (v) 30-carbon lanosterol, the first sterol, formed by the cyclisation of squalene-2,3S-epoxide. Each of the genes coding each of the enzymes in the cholesterol biosynthesis pathway has a sterol regulatory (or response) element (SRE) in its promotor, and is activated by the nuclear form of the master transcription factor sterol regulatory element-binding protein-2 (SREBP-2; Horton et al., 2002, 2003; Goldstein et al., 2006; Mazein et al., 2013; Brown et al., 2018). The low-density lipoprotein (LDL) receptor gene also has a SRE and is regulated by SREBP-2.

### SREBP-2 Pathway

**Figure 2:**
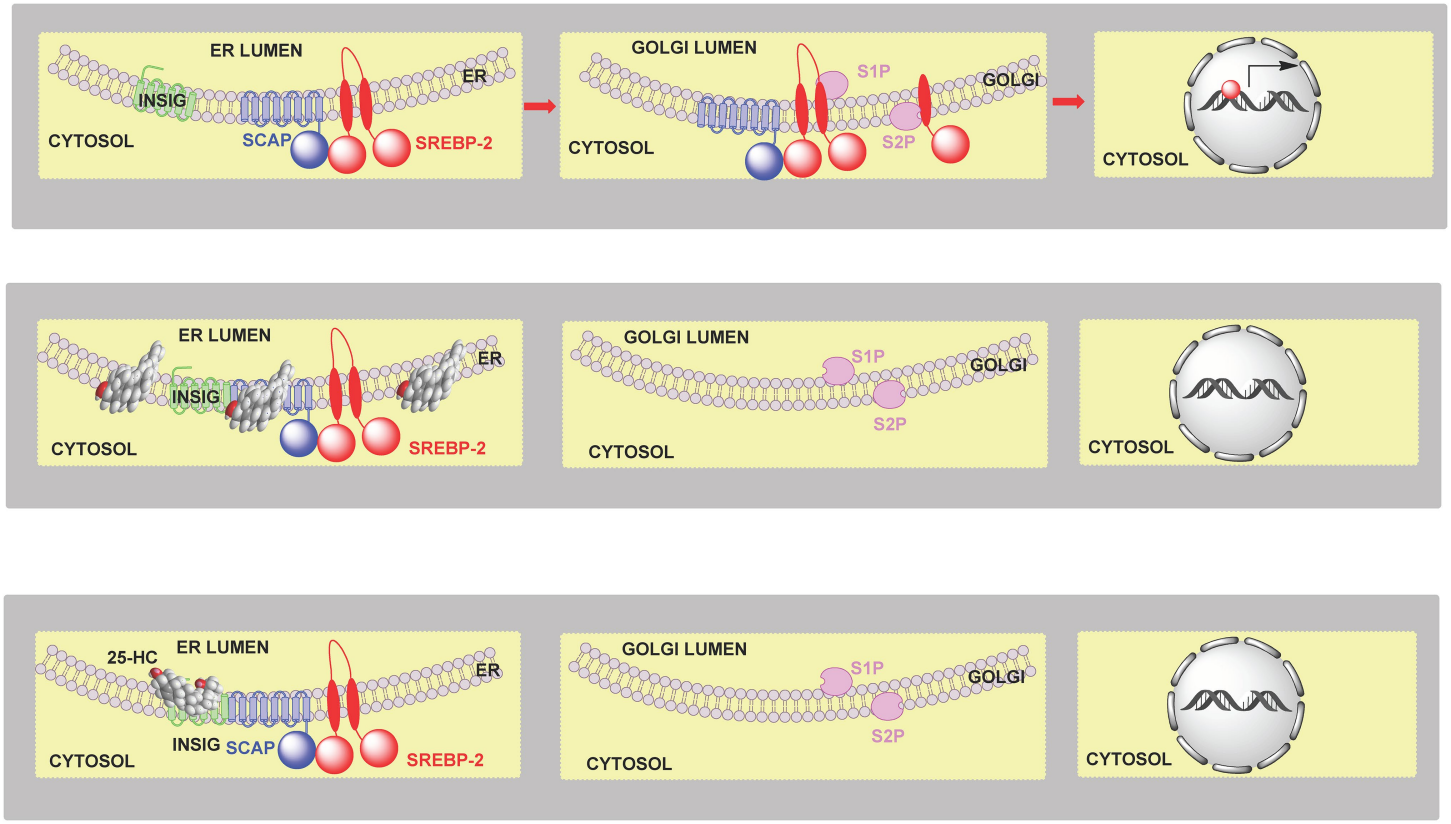
Cartoon representation of the regulation of cholesterol biosynthesis by SREBP-2. **Upper panel**, left to right, SCAP transports SREBP-2 from the endoplasmic reticulum to the Golgi, it is then processed to the active nuclear form which translocates to the nucleus and activates target gene transcription. **Central panel**, when cholesterol levels are elevated (>5 mole % endoplasmic reticulum lipids), cholesterol binds to SCAP, which then binds to INSIG retaining the SCAP-SREBP-2 complex in the endoplasmic reticulum preventing transport to, and formation of the active transcription factor, in the Golgi. **Lower panel**, 25-HC can bind to INSIG tethering the SCAP-SREBP-2 complex in the endoplasmic reticulum and restraining SREBP-2 transport and processing. SREBP-2 target genes include *ACAT2*, *HMGCS*, *HMGCR*, *MVK*, *PMVK*, *MVD*, *IDI1*, *GGPS1*, *FDPS*, *FDT1*, *SQLE*, *LSS*, *CYP51A1*, *TM7SF2*, *MSMO1 (SC4MOL)*, *NSDHL*, *HSD17B7*, *EBP*, *SC5D*, *DHCR7*, *DHCR24*, *LDLR*, *INSIG1*, and *STARD4* (Horton et al., 2002, 2003).

High levels of cellular cholesterol lead to elevated cholesterol levels in the endoplasmic reticulum (i.e., >5 mole % endoplasmic reticulum lipids; Radhakrishnan et al., 2008; Das et al., 2014; Infante and Radhakrishnan, 2017) and to a process termed convergent inhibition whereby cholesterol synthesis and expression of the LDL-receptor are reduced restoring cholesterol to its optimal level (Horton et al., 2002; Goldstein et al., 2006; Brown et al., 2018). SREBP-2 is synthesised in the endoplasmic reticulum and immediately binds to the transport protein SCAP (SREBP cleavage-activating protein) which, in the absence of elevated levels of cholesterol transports SREBP-2 to the Golgi where it undergoes two cleavage reactions catalysed by the serine protease S1P (site 1 protease) and the metalloprotease S2P (site 2 protease) to release the active transcription factor that translocate to the nucleus and activates target gene expression (Figure 2, upper panel). Mammalian cells produced three SREBP isoforms SREBP-1a, SREBP-1c and SREBP-2. SREBP-1a and SREBP-1c are produced by the same gene by the use of different promotors and alternative splicing. In the liver the dominant SREBPs are SREBP-1c and SREBP-2, where SREBP-2 is primarily involved in stimulating cholesterol synthesis while SREBP-1c primarily stimulates fatty acid synthesis (Horton et al., 2002). SREBP-2 also stimulates expression of the LDL-receptor and the endoplasmic reticulum resident protein INSIG (insulin induced gene; Goldstein et al., 2006). When cholesterol levels are elevated in the endoplasmic reticulum [>5 mole % endoplasmic reticulum lipids (Radhakrishnan et al., 2008; Das et al., 2014; Infante and Radhakrishnan, 2017)], cholesterol binds to SCAP which in turn binds to INSIG tethering the SCAP-SREBP-2 complex in the endoplasmic reticulum and preventing transport to the Golgi, and thus SREBP-2 proteolysis and activation (Figure 2, central panel). In this way cholesterol regulates its own biosynthesis and uptake by the LDL-receptor (Figure 2).

### LDL-Receptor, Cholesterol Uptake, and Intracellular Transport

**Figure 3:**
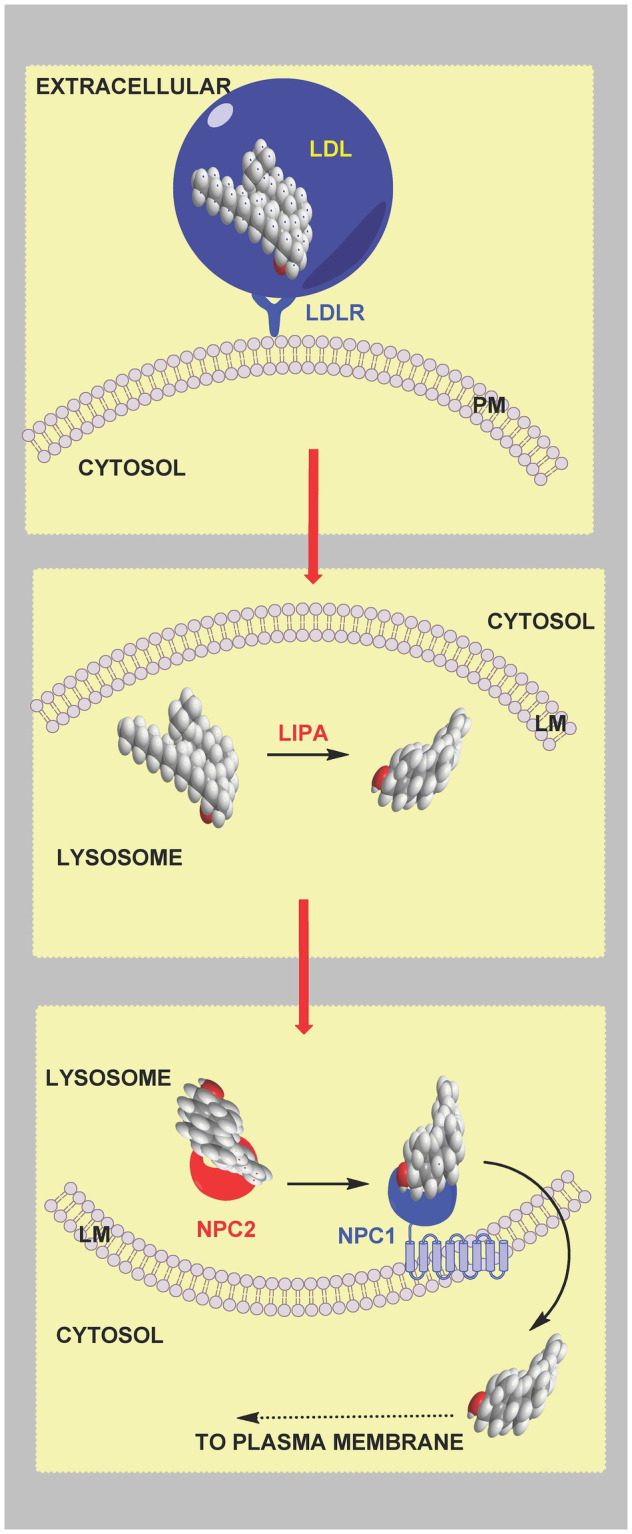
Cartoon representation of cholesterol uptake and transport from the lysosome. LDL particles bind to the LDL-receptor (LDLR) on the extracellular surface of the plasma membrane and are taken up by receptor mediated endocytosis. Endosomes combine with the lysosome where cholesterol esters are hydrolysed by LIPA. Non-esterified cholesterols is transported by soluble NPC2 and membrane bound NPC1 to the lysosomal membrane for export. Exported cholesterol is thought to travel first to the plasma membrane before reaching the endoplasmic reticulum.

Cells expressing the LDL-receptor take-up cholesterol by a process called receptor mediated endocytosis (Brown and Goldstein, 1986), where LDL particles bind to the LDL receptor located on the plasma membrane and become internalised in endosomes and degraded in the lysosome. Cholesterol esters are hydrolysed by lysosomal acid lipase (*LIPA*; Goldstein et al., 1975; Brown et al., 1976) and non-esterified cholesterol transferred to the lysosomal membrane and ultimately out of the lysosome by the combined action of Niemann-Pick proteins NPC2 and NPC1 (Figure 3; Kwon et al., 2009; Li et al., 2016; Infante and Radhakrishnan, 2017). In this way a deficiency in endoplasmic reticulum cholesterol is ultimately restored.

**Figure 4:**
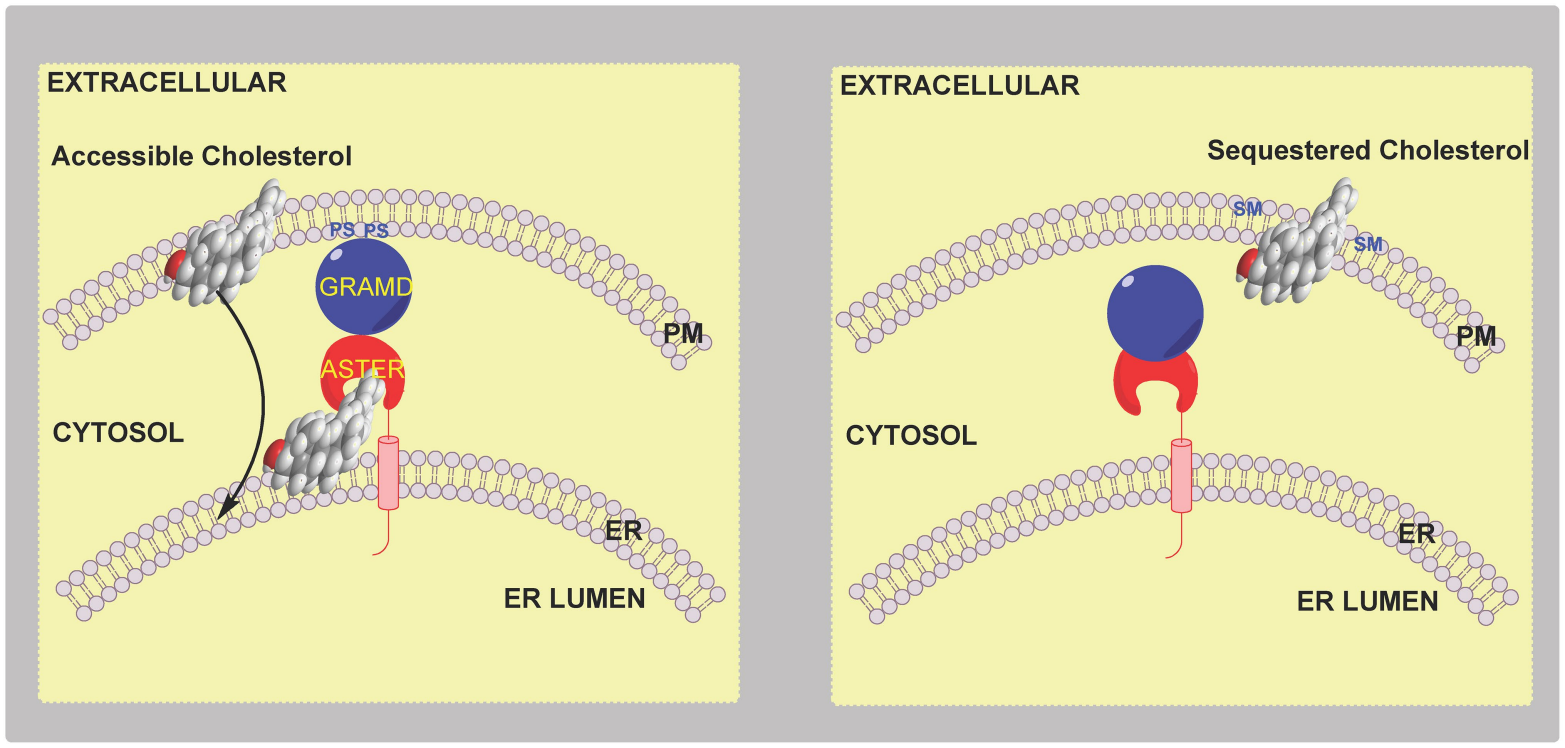
Cartoon illustration of the involvement of Aster proteins in cholesterol transport and regulation. **Left panel**, an elevation in accessible cholesterol leads to presentation of PS on the inner leaflet of the plasma membrane and formation of a membrane contact site *via* the GRAM domain of the endoplasmic reticulum resident protein Aster. Accessible cholesterol is then transferred by the ASTER domain of the protein from the plasma membrane to the endoplasmic reticulum to be sensed by the SREBP-machinery. **Right panel**, when accessible cholesterol in the plasma membrane is below a critical level there is no binding of the Aster protein to the plasma membrane.

However, there is a conundrum, most of the cell’s cholesterol is found in the plasma membrane, while the proteins SREBP-2, SCAP and INSIG are in the endoplasmic reticulum. What’s more cholesterol released from the lysosome is believed to travel to the plasma membrane prior to the endoplasmic reticulum (Das et al., 2014; Infante and Radhakrishnan, 2017; Trinh et al., 2020). So how can plasma membrane levels of cholesterol be sensed by proteins located in the endoplasmic reticulum? The answer lies in the concept of “accessible” plasma membrane cholesterol (Das et al., 2014). Three pools of plasma membrane cholesterol have been suggested. A pool of *cholesterol sequestered by sphingomyelin* (SM) comprising about 15 mole % of plasma membrane lipids, a second pool *sequestered in some other way* comprising about 12 mole % of plasma membrane lipids and essential to maintain membrane morphology, and an *accessible pool of cholesterol* comprising about 16 mole % of plasma membrane lipids in cholesterol replete cells that signals to the regulatory machinery of the endoplasmic reticulum (Das et al., 2014). The concept of accessible cholesterol is derived from “thermodynamic activity” of species in a condensed phase, where activity, or chemical potential, is dependent on environment. It appears that small changes in the level of accessible cholesterol which are insufficient to cause a measurable change in the total membrane cholesterol are sufficient to be sensed by the SREBP-machinery and regulate cholesterol homeostasis (Das et al., 2014; Infante and Radhakrishnan, 2017). Recent studies indicate that the level of the accessible cholesterol pool dictates the formation of membrane contact sites between the plasma membrane and endoplasmic reticulum through the family of sterol transport proteins called Asters coded by *GRAMD1* genes (Sandhu et al., 2018; Naito et al., 2019; Ferrari et al., 2020; Trinh et al., 2020; Xiao et al., 2021). Aster proteins have (i) a GRAM domain that binds to the plasma membrane in a manner dependent on accessible cholesterol and also glycerolphosphoserine (PS), (ii) a central fold (ASTER domain) resembling the sterol binding fold in StARD (steroidogenic acute regulatory) proteins and (iii) a C-terminal transmembrane domain (Sandhu et al., 2018; Naito et al., 2019; Ferrari et al., 2020; Trinh et al., 2020). When the accessible pool of cholesterol expands, e.g., following cholesterol uptake by the LDL-receptor and transport through the lysosomal system, there is a change in plasma membrane presentation of PS resulting in Aster binding through the GRAM domain and cholesterol transport *via* the central ASTER domain to the endoplasmic reticulum (Figure 4). In this way small changes in total plasma membrane and cellular cholesterol are detected by the endoplasmic reticulum sterol regulatory machinery which then provides a mechanism for restoration of the disturbance, rapidly protecting the cell from cholesterol overload.

### High Density Lipoprotein Particles and Reverse Cholesterol Transport

Reverse cholesterol transport (RCT) is the mechanism by which cholesterol is returned to the liver from peripheral tissues *via* high density lipoprotein (HDL) particles. Non-esterified cholesterol is transported across the plasma membrane to apolipoprotein A1 (ApoA1) or pre-β HDL by ATP-binding cassette transporters ABCA1 and ABCG1, two translation products of liver X receptor (LXR) target genes (see section Liver X Receptors), expression of which is activated by oxysterol binding to heterodimers of LXR and retinoid X receptor (RXR; Venkateswaran et al., 2000; Evans and Mangelsdorf, 2014; Wang and Tontonoz, 2018). Free cholesterol then becomes esterified in HDL particles by lecithin cholesterol acyl transferase (LCAT) with the acyl donor derived from position 2 of a glycerophosphocholine. LXR target genes also include apolipoproteins and proteins involved in lipoprotein remodelling further demonstrating a link between LXRs and RCT (Wang and Tontonoz, 2018). Significantly, mutations in *ABCA1* can lead to Tangier disease, an inborn error of metabolism characterised by reduced plasma HDL cholesterol and accumulation of cholesterol in peripheral tissue (Rust et al., 1999).

Scavenger receptor class B member 1 (SR-B1) acts as the major receptor for HDL cholesterol (Acton et al., 1996). It is abundant in the liver and steroidogenic tissue, and facilitates the selective uptake of cholesterol from HDL (Glass et al., 1983). In the liver surplus cholesterol is converted into bile acids or excreted into bile. Unlike the situation with the LDL-receptor, little is known about the pathway taken by HDL-derived cholesterol beyond SR-B1 mediated uptake. However, Aster proteins may again be the link between accessible cholesterol, this time HDL-derived, at the plasma membrane and the endoplasmic reticulum. In fact, Aster-B is enriched in steroidogenic tissue and its expression is required for storage of HDL-derived cholesterol and steroidogenisis in the adrenal cortex (Sandhu et al., 2018).

### Accessible Cholesterol

**Figure 5:**
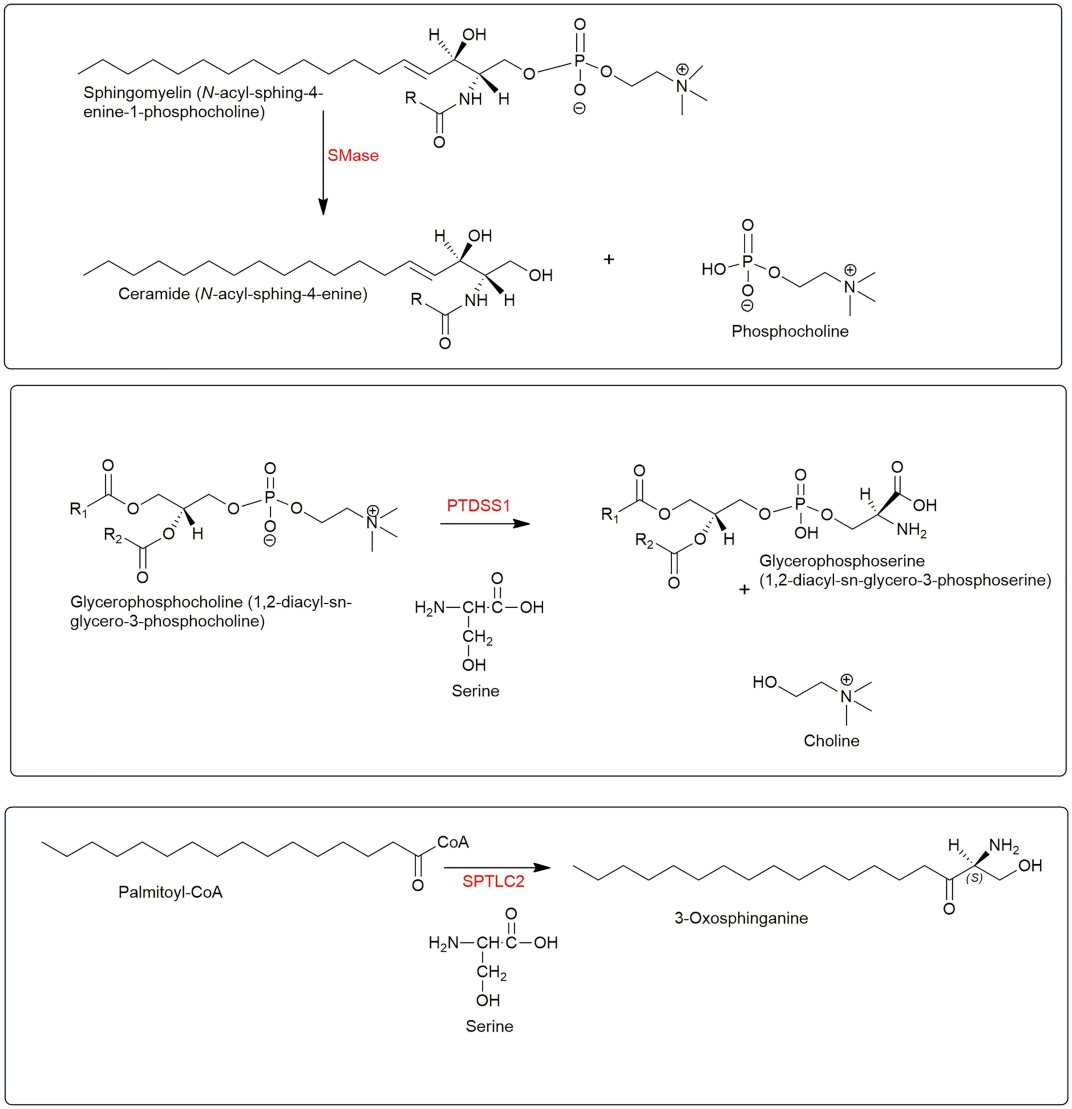
Lipidomoics of accessible cholesterol. **Upper panel**, SMase hydrolyses SM to ceramide and phosphocholine. **Central panel**, PTDSS1 catalyses the conversion of PC to PS. **Lower panel**, SPTLC2 catalyses the formation of 3-oxosphinganine from palmitoyl-CoA and serine.

The involvement of membrane phospholipids in cholesterol transport from the plasma membrane to the endoplasmic reticulum is intriguing and emphasises that a true lipidomic approach is required to unravel the secrets of cell biology. In early studies where the concept of accessible cholesterol was proposed a pool of SM sequestered cholesterol was defined. The difference in accessible and sequestered pools being in the thermodynamic chemical activity, or chemical potential, of cholesterol as a consequence of its membrane environment. When cholesterol is in the regulatory and accessible pool it is available to bind bacterial pore-forming toxins, e.g., perfringolysin O, PFO; anthrolysin ALO (Das et al., 2013, 2014; Infante and Radhakrishnan, 2017), and cholesterol from the SM sequestered pool only becomes accessible to pore-forming toxins after treatment with sphingomyelinase (SMase) at which point it becomes accessible for movement to the endoplasmic reticulum (Das et al., 2014). SMase hydrolyses SM to phosphocholine and ceramide (Figure 5). The residual pool of cholesterol does not bind pore forming toxins even after SMase treatment but is essential to maintain membrane morphology (Das et al., 2014).

As mentioned above the GRAM domain of the endoplasmic reticulum resident Aster proteins binds to PS in the plasma membrane in response to cholesterol accumulation, the Aster domain with its StARD-like fold then extracts cholesterol and moves it to the endoplasmic reticulum (Figure 4; Sandhu et al., 2018; Ferrari et al., 2020). The reason why GRAM domains recognise PS only in the presence of excess accessible cholesterol is unknown, however, one hypothesis is that an increase in the accessible pool of cholesterol alters the presentation of PS in the inner leaflet of the plasma membrane and facilitates GRAM domain binding and subsequent ASTER domain cholesterol transfer to the endoplasmic reticulum (Ferrari et al., 2020). Importantly, cells deficient in phosphatidylserine synthase 1 (PTDSS1), an enzyme that catalyses the conversion of glycerophosphocholine (PC) to PS (Figure 5, central panel), and which are lacking in PS fail to transport LDL-derived cholesterol to the endoplasmic reticulum, which instead accumulates as accessible cholesterol in the plasma membrane (Trinh et al., 2020). This data confirmed the hypothesis that LDL-derived cholesterol leaving the lysosome first moves to the plasma membrane where it expands the pool of accessible cholesterol before moving to the endoplasmic reticulum and inhibiting SREBP-2 processing (Trinh et al., 2020).

Remarkably SM and PS have opposite effects on cholesterol transport from the plasma membrane. SM is concentrated in the outer leaflet and sequesters cholesterol while PS is concentrated in the inner leaflet and is essential for cholesterol movement to the endoplasmic reticulum.

### Cholesterol Precursors and Metabolites

**Figure 6:**
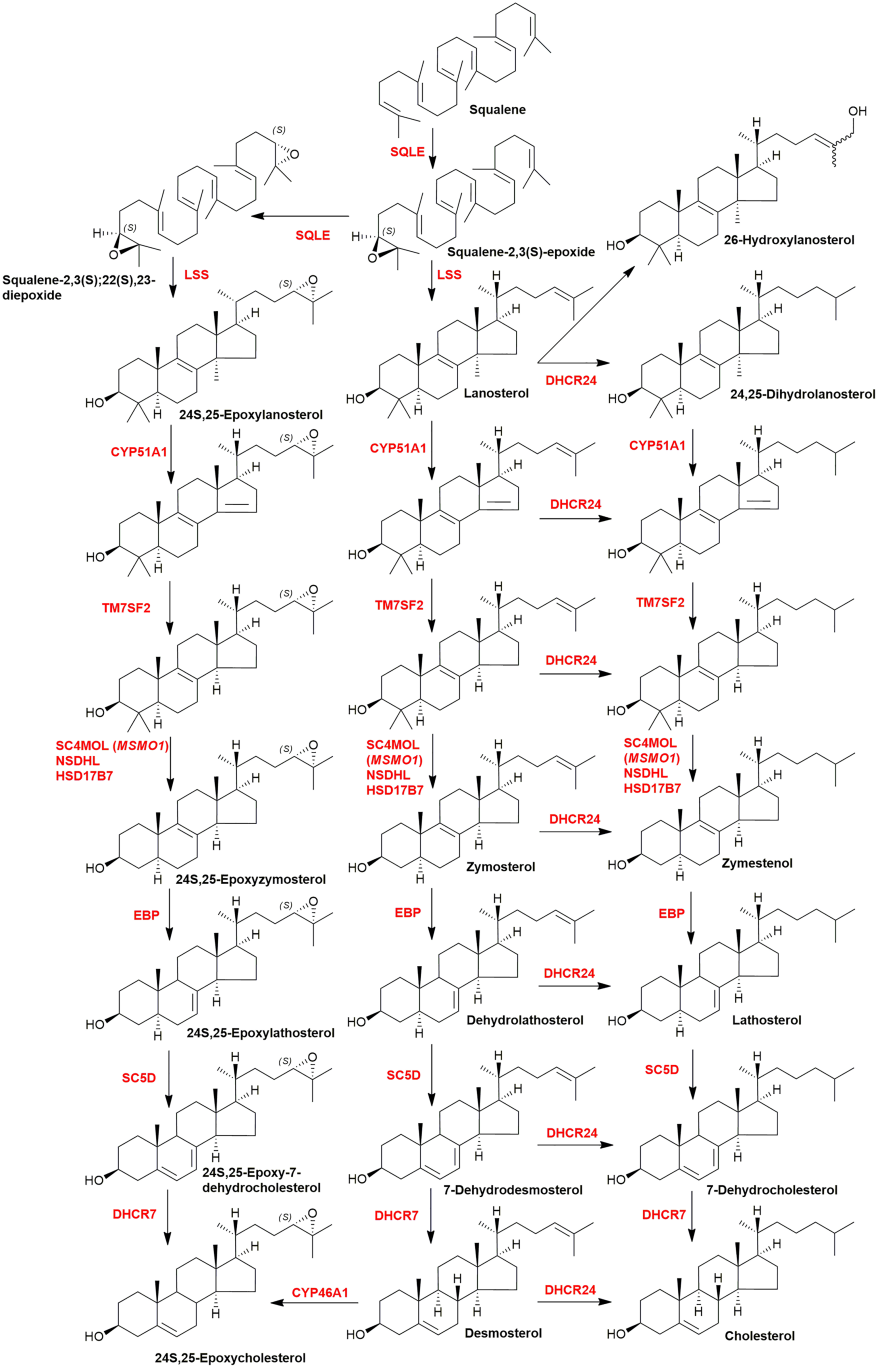
Pathway from squalene to cholesterol and 24S,25-epoxycholesterol. Enzymes are indicated in red.

**Figure 7:**
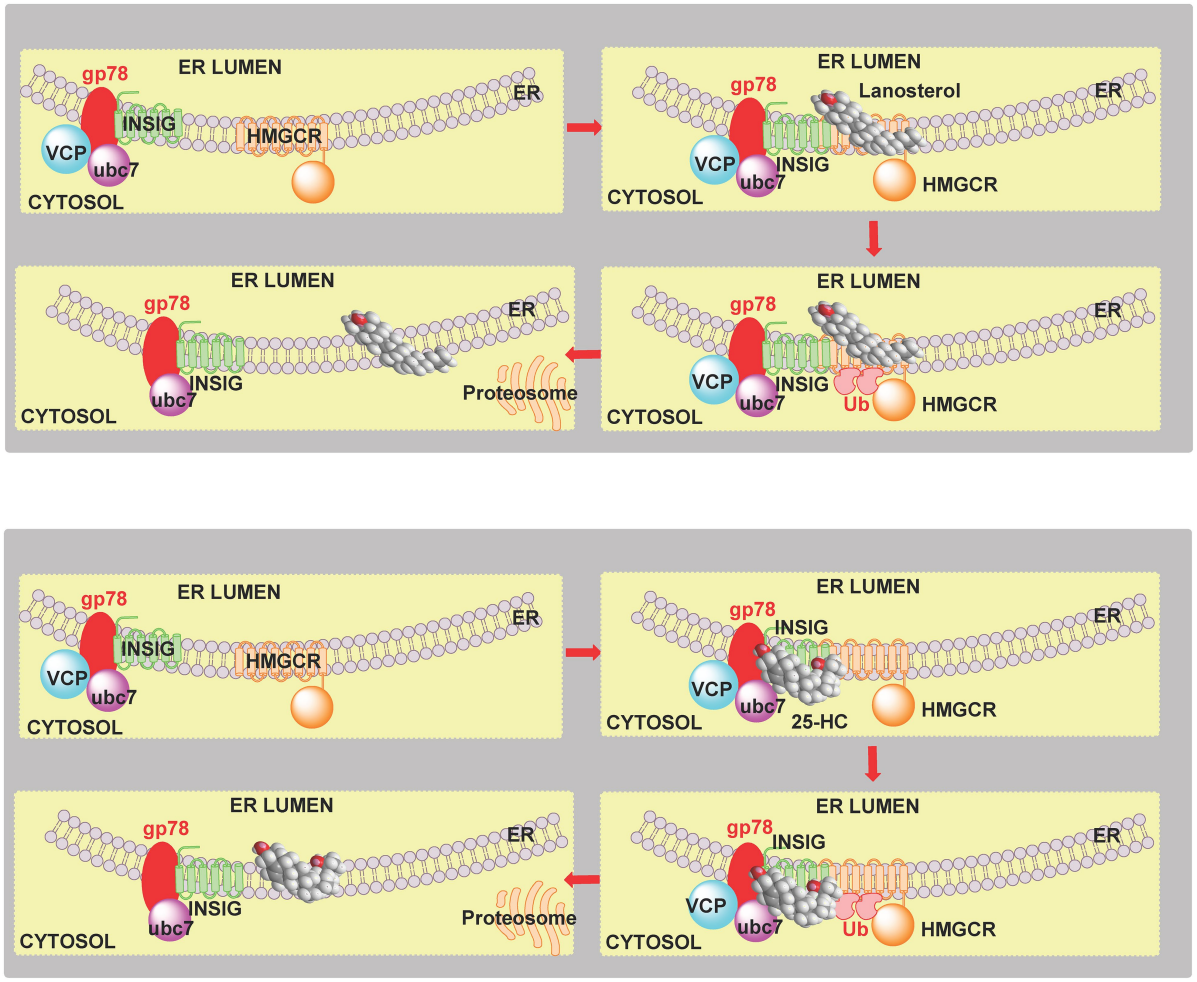
Cartoon representation of the sterol-induced degradation of HMG-CoA reductase. **Upper panel**, 4,4-dimethyl sterols induce the binding of INSIG to HMG-CoA reductase (HMGCR). The E3 ubiquitin ligase gp78 and E2 ubiquitin-conjugating enzyme ubc7 ubiquitinate (Ub) HMG-CoA reductase. In a final step stimulated by geranylgeraniol, ubiquitinated HMG-CoA reductase is extracted from the membrane by VCP and delivered to the proteosome. **Lower panel**, 25-HC similarly induces degradation of HMG-CoA reductase, however, it has been suggested that sterol binding is to INSIG rather than the reductase (Goldstein et al., 2006).

The SREBP-2 pathway is not only regulated by cholesterol but also by its precursors and metabolites (Radhakrishnan et al., 2007; Spann et al., 2012; Chen et al., 2019). Like cholesterol, desmosterol binds to SCAP and prevents the activation of SREBP-2 to its nuclear form (Radhakrishnan et al., 2007; Spann et al., 2012). Oxysterols when the added oxygen function is on the side-chain (side-chain oxysterols) also inhibit processing of SREBP-2 to its active form but by binding to INSIG rather than SCAP but still tethering the SCAP-SREBP-2 complex in the endoplasmic reticulum thereby preventing transport of SREBP-2 to the Golgi for activation to its nuclear form (Figure 2, lower panel; Radhakrishnan et al., 2007). An alternative route by which the cholesterol precursor lanosterol and side-chain oxysterols regulate cholesterol biosynthesis is *via* degradation of HMG-CoA reductase (Song et al., 2005; Goldstein et al., 2006; Chen et al., 2019). Lanosterol and other 4,4-dimethyl sterols (Figure 6) trigger the binding of HMG-CoA reductase to INSIG, this leads to the membrane-embedded E3 ubiquitin ligase gp78 along with the recruited E2 ubiquitin-conjugating enzyme ubc7 to ubiquitinate the reductase which then is extracted from membrane by the ATPase VCP and delivered by to the proteosome for degradation (Figure 7, upper panel; Goldstein et al., 2006). The final step is stimulated by geranylgeraniol. The side-chain oxysterol 25-hydroxycholesterol (25-HC) also triggers the binding of HMG-CoA reductase to INSIG (Sever et al., 2003) and its subsequent degradation, and it has been suggested that 25-HC mediates this effect by binding to INSIG, while 4,4-dimethyl sterols may bind to the reductase (Radhakrishnan et al., 2007). Interestingly 27-hydroxylanosterol [more correctly named as (*E/Z*)26-hydroxylanosterol or lanosta-8(*E*),24(*E/Z*)-diene-3β,26-diol (Fakheri and Javitt, 2012)] has equivalent activity to lanosterol but at an order of magnitude lower concentration (Song et al., 2005). Lanosterol does not regulate the SREBP-2 pathway, but other 4,4-dimethyl sterols will repress the processing of SREBP-2 and also stimulate HMG-CoA reductase degradation (Chen et al., 2019).

Besides down-regulation the SREBP-2 pathway cells are protected from cholesterol overload by the endoplasmic reticulum enzyme acyl-CoA cholesterol acyl transferase (ACAT, sterol O-acyltransferase 1, SOAT1) which esterifies cholesterol for storage in lipid droplets. Interestingly, the cholesterol-derived oxysterol, 25-HC, stimulates esterification of cholesterol to its esterified form (Chang et al., 1997; Du et al., 2004).

### Liver X Receptors

**Figure 8:**
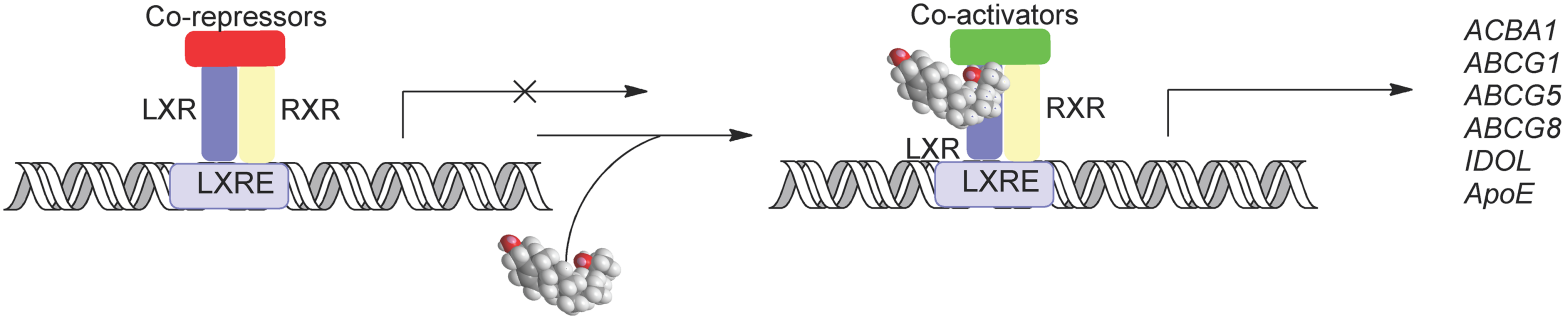
Activation of LXRs by oxysterol. In the absence of ligand LXR-RXR heterodimers bind to the LXR response element (LXREs) and recruit co-repressors and supress gene expression. When activated by oxysterols co-repressors are replaced by co-activators leading to the expression of LXR target genes.

**Figure 9:**
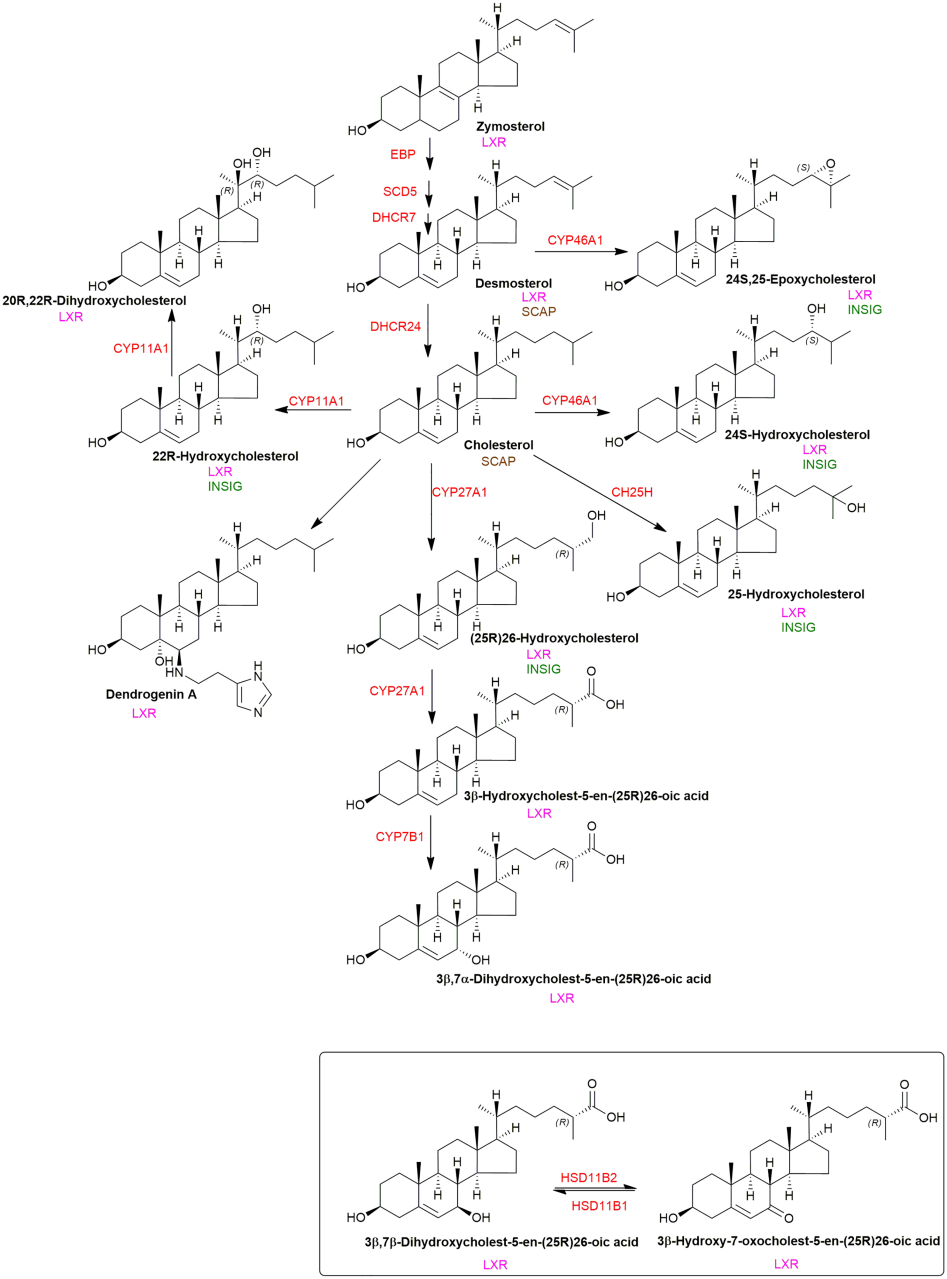
Crosstalk between ligands to LXR, INSIG and SCAP. Enzymes are shown in red. LXR, INSIG and SCAP binding is illustrated where appropriate.

The liver X receptors (LXRα, NR1H3; LXRβ, and NR1H2) are oxysterol receptors (Figure 8; Janowski et al., 1996, 1999; Forman et al., 1997; Lehmann et al., 1997; Fu et al., 2001; Theofilopoulos et al., 2013; Maqdasy et al., 2016). Other endogenous ligands include the cholesterol precursors zymosterol and desmosterol (Yang et al., 2006), C_27_ cholestenoic acids (Song and Liao, 2000; Ogundare et al., 2010; Theofilopoulos et al., 2014), and dendgrogenin A, the histamine conjugate of 5α,6α-epoxycholesterol (Figure 9; Segala et al., 2017). The target genes of LXRs code for the cholesterol export proteins ABCA1 and ABCG1, the cholesterol carrier ApoE, the inducible degrader of the LDL-receptor (IDOL), SREBP-1c, and in mouse CYP7A1, the enzyme which catalyses the rate-determining step of the neutral pathway of bile acid biosynthesis (Laffitte et al., 2001; Joseph et al., 2002; Zelcer and Tontonoz, 2006; Zelcer et al., 2009; Wang and Tontonoz, 2018). In addition, the *Gramd1b* gene coding the Aster-B protein is a direct transcriptional target of the LXRs (Sandhu et al., 2018). In combination, activation of LXRs has the overall effect of reducing cellular cholesterol levels. There is considerable cross-talk between SREBP and LXR regulated pathways starting at the level of oxysterols where many side-chain oxysterols activate both LXR and inhibit SREBP processing *via* binding to INSIG (Figures 2, 9; Janowski et al., 1999; Radhakrishnan et al., 2007).

## Interferons, Cholesterol 25-Hydroxylase and Accessible Cholesterol in Protection Against Microbial Pathogens

### Interferons and 25-HC

IFNs (IFNs) are cytokines involved in the communication between cells which trigger defence mechanism of the immune system against pathogens including viruses (Blanc et al., 2011, 2013; Liu et al., 2013; Cyster et al., 2014) and bacteria (Bauman et al., 2009; Diczfalusy et al., 2009; Cyster et al., 2014). Type I IFNs, e.g., IFNβ, are produced by many types of cell including macrophages, fibroblasts and endothelial cells and bind to the cell surface IFNα/β receptor (IFNAR) on target cells, while type II IFN (IFNγ in human) produced by T-cells binds to the IFNGR on target cells.

The Toll-like receptor 4 (TLR4) is a pattern recognition receptor (PRR) which is activated by lipopolysaccharide (LPS), a cell wall component of many Gram-negative and some Gram-positive bacteria and also by some viral proteins. Over the last decade links between TLR4, TLR3 (an intracellular PRR), bacterial and viral infections, IFNs, and the oxysterol 25-HC have been uncovered. Activation of macrophage or dendritic cells (antigen-presenting cell) by TLR4 or TLR3 ligands leads to induction of IFNβ, activation of IFNAR and up-regulation of *Ch25h* (*CH25H* in human) the gene encoding cholesterol 25-hydroxylase leading to elevated levels of plasma 25-HC (Bauman et al., 2009; Diczfalusy et al., 2009; McDonald and Russell, 2010; Park and Scott, 2010; Blanc et al., 2013; Liu et al., 2013). *Ch25h*/*CH25H* is now classified as an interferon stimulated gene. 25-HC can be regarded as an immunoregulatory oxysterol; it has a role in the adaptive immune system, suppressing the production of immunoglobulin A (IgA) by B cells (Bauman et al., 2009), it also restrains interleukin-1β (IL-1β) driven inflammation in macrophages by preventing AIM2 (absent in melanoma 2) inflammasome activation (Dang et al., 2017). This effect is achieved by restricting cholesterol biosynthesis by inhibiting the processing of SREBP-2 (Dang et al., 2017). High cholesterol synthesis is required AIM2-dependent inflammasome activation and IL-1β release by macrophages.

### Cholesterol-Dependent Cytolysins, Accessible Cholesterol and 25-HC

**Figure 10:**
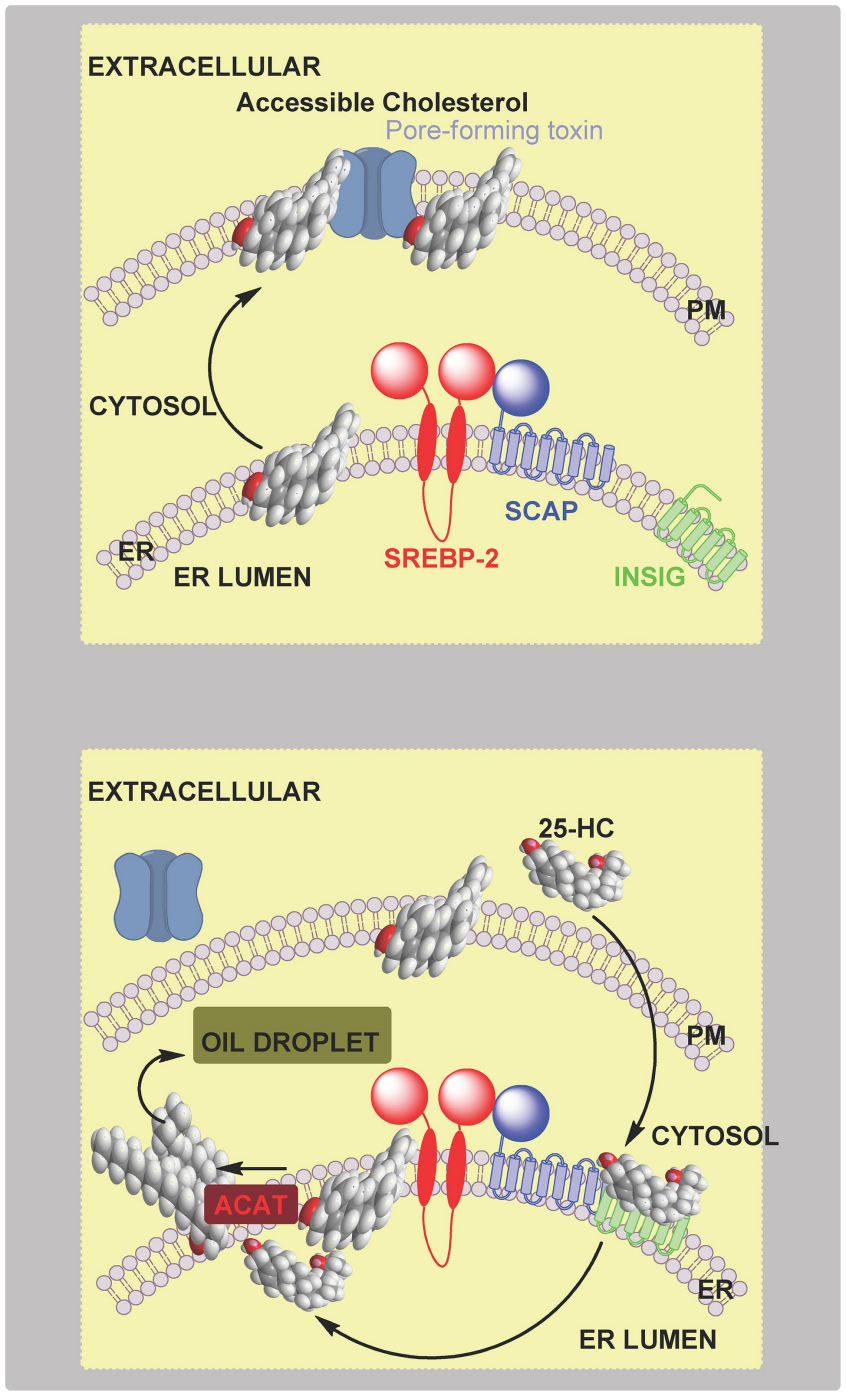
Cartoon representation of the protection by 25-HC of macrophages and neutrophiles against pore-forming toxins. **Upper panel**, pore-forming toxin binds to accessible cholesterol in the plasma membrane, oligomerises and create a pore, ultimately leading to cell death. **Lower panel**, 25-HC generated by macrophages in response to infection rapidly crosses the cell membrane, and (i) inhibits SREBP-2 processing leading to reduced cholesterol synthesis and (ii) activates ACAT/SOAT and cholesterol esterification, in combination leading to a reduction in plasma membrane accessible cholesterol. The consequence is reduced binding of pore-forming toxins to the plasma membrane and protection of the cell. 25-HC can also activate LXR to enhance cholesterol export and can encourage the ubiquitination and degradation of HMG-CoA reductase to repress cholesterol synthesis (not shown).

PFO is a pore-forming toxin secreted by the Gram-positive bacteria *Clostridium perfringens* and is a member of a family of cholesterol-dependent cytolysins (CDC). A mutated version of PFO, PFO*, binds to accessible cholesterol, and at 4°C does not form pores and kill cells (Das et al., 2013). ALO is a closely related CDC, and like PFO*, its cholesterol binding sub-domain ALOD4, has been used extensively to assess the accessible cholesterol content of membranes (Das et al., 2013, 2014; Infante and Radhakrishnan, 2017; Naito et al., 2019; Ferrari et al., 2020; Trinh et al., 2020). Remarkably, 25-HC protects macrophages and neutrophiles against CDCs including PFO demonstrating an acute interplay between oxysterols and accessible cholesterol (Zhou et al., 2020). The mechanism behind this protection proceeds through activation of PRR on macrophages, IFN-induced expression of the enzyme CH25H and production of 25-HC (Zhou et al., 2020). As discussed above, 25-HC can (i) reduce cholesterol synthesis *via* binding to INSIG and prevent processing of SREBP-2 to its active form; (ii) induce the degradation of HMG-CoA reductase (Radhakrishnan et al., 2007); (iii) activate the LXRs and induce cholesterol export (Wang and Tontonoz, 2018) and mediate cholesterol ester formation by activation of ACAT/SOAT (Du et al., 2004). It is possible that all four effects contribute to 25-HC mediated protection against CDCs by reducing the pool of accessible cholesterol in the macrophage/neutrophile plasma membrane, and thus lead to reduced CDC binding and pore formation, ultimately reducing CDC toxicity (Figure 10; Zhou et al., 2020). Current data suggest that reduction in cholesterol biosynthesis is most important in reducing plasma membrane accessible cholesterol and that, surprisingly, IFN will increase cholesterol ester formation even in the absence of *Ch25h* (Zhou et al., 2020). By maintaining a low pool of accessible cholesterol macrophages and neutrophiles are protected against CDCs, it is likely that 25-HC will also protect endothelium and epithelium cells against pathogen produced toxins *via* a similar mechanism.

### Bacterial Infection, Accessible Cholesterol and 25-HC

**Figure 11:**
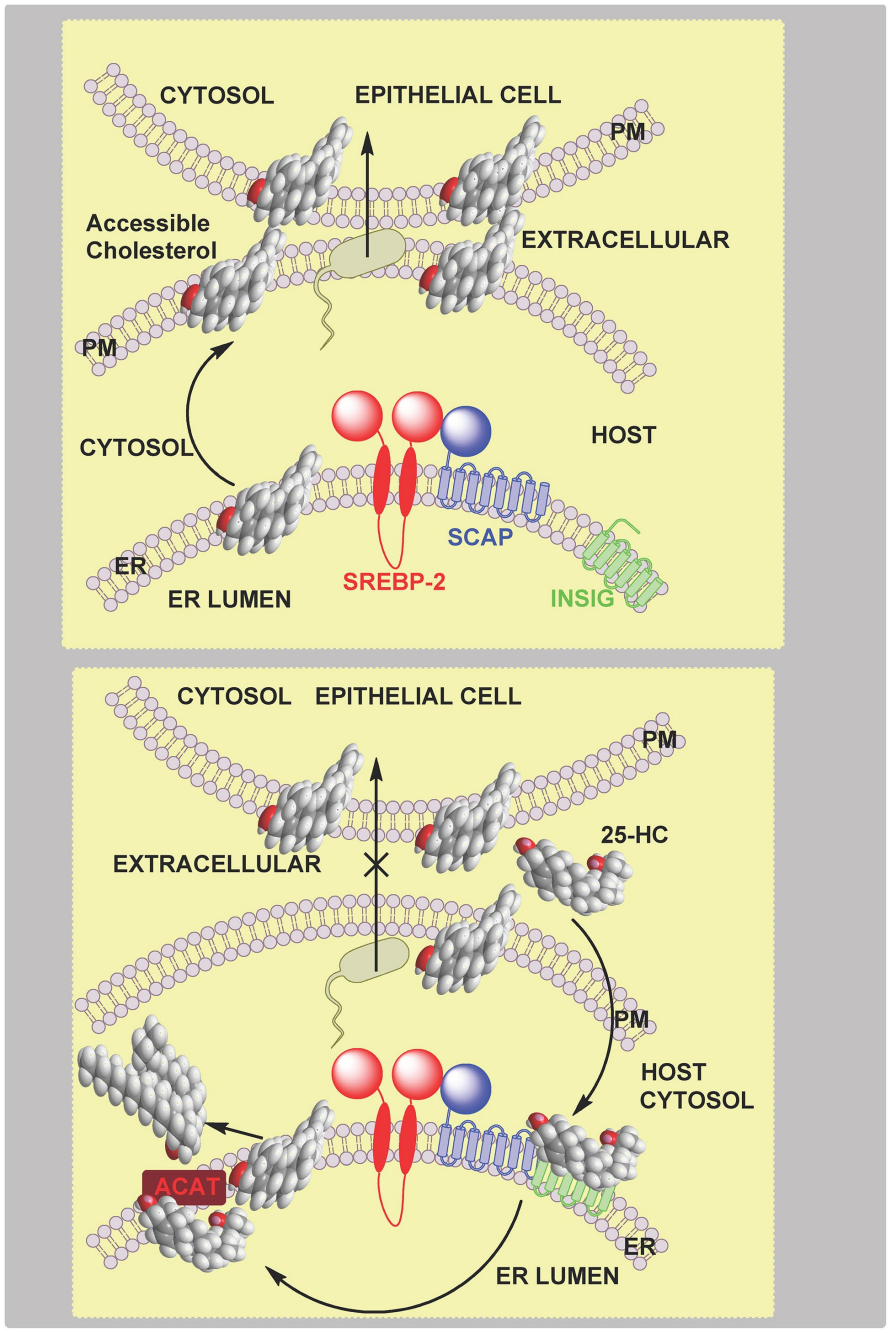
Cartoon representation of the inhibition by 25-HC of bacterial spread between epithelial cells. **Upper panel**, plasma membrane accessible cholesterol is required for bacterial cell–cell transmission. **Lower panel**, activated macrophages secrete 25-HC which stimulates the endoplasmic reticulum-located enzyme ACAT/SOAT to rapidly esterify cholesterol for storage in lipid droplets leading to reduced accessible cholesterol in the plasma membrane, this prevents bacterial spread across neighboring cells. A supporting role in reducing accessible cholesterol is played by inhibition of the SREPB-2 pathway but over a longer timeframe.

The mucosal epithelium provides a physical barrier between microbial communities and underlying tissue of the host. One way in which the host is protected against microbes is through IFNγ-activated macrophages which communicate with epithelium cells to clear local infections. *CH25H* is one of the hundreds of IFN-stimulated genes which are bactericidal, and it has been shown recently that the enzymatic product of the expressed gene, 25-HC, can prevent in a paracrine fashion, cell–cell transmission in the infected host (Abrams et al., 2020). 25-HC can suppress the contact-dependent cell to cell spread of *Listeria monocytogenes*, a model enteric pathogen, in epithelial tissue. Using ALOD4 to distinguish between pools of plasma membrane cholesterol, 25-HC was found to deplete accessible cholesterol and inhibit bacterial spread and infection, leading to the conclusion that accessible cholesterol is required for bacteria to penetrate adjacent cells (Abrams et al., 2020). Like *L. monocytogenes*, *Shigella flexneri* undergoes cell-to-cell spread *via* membrane protrusions, and like *L. monocytogenes* its spread is inhibited by 25-HC reducing host-cell plasma membrane accessible cholesterol (Abrams et al., 2020). The mechanism by which 25-HC reduces accessible cholesterol in the host epithelium cell was suggested to be *via* activation of endoplasmic reticulum-located ACAT/SOAT, triggering rapid internalisation of accessible cholesterol from the plasma membrane (Abrams et al., 2020). This conclusion was based on data showing inhibition of ACAT/SOAT prevents the removal of accessible cholesterol by the LXR and INSIG ligands 20S-hydroxycholesterol (20S-HC), 25-HC and (25R)26-hydroxycholesterol (26-HC, more commonly referred to as 27-HC; Abrams et al., 2020), however, this data does not rule out LXR activation and inhibition of SREBP-2 processing playing a supporting role in the defence against bacterial spread over a longer time period (Figure 11).

### Antiviral Activity of 25-HC and Other Oxysterols

25-HC has been shown to have broad antiviral activity against enveloped (Blanc et al., 2013; Liu et al., 2013; Chen et al., 2014) and nonenveloped viruses (Doms et al., 2018; Shawli et al., 2019). In early studies it was shown that in response to viral infection macrophage generated IFN activates the expression of *Ch25h* with the resultant generation and secretion of 25-HC providing both a paracrine and autocrine antiviral response (Blanc et al., 2011, 2013; Liu et al., 2013). This can be achieved by down-regulation of the host cholesterol biosynthesis pathway, consistent with 25-HC repressing the processing of SREBP-2 (Blanc et al., 2013). It is noteworthy, that in one of the early studies performed mostly with mouse cytomegalovirus (MCMV) and bone-marrow-derived macrophages (BMDM) the antiviral activity of 25-HC was increased in lipid depleted conditions where the SREBP-2 pathway is active (Blanc et al., 2013). 25-HC will reduce both the *de novo* synthesis of cholesterol and receptor mediated up-take *via* the LDL-receptor. However, in a different cell model, it was found that over expression of *Srebp-2* in HEK293T cells did not reverse the antiviral effect of 25-HC and instead it was suggested that the antiviral activities of 25-HC were through changing cell membrane properties to inhibit membrane fusion, a requirement to release viral genetic material for subsequent replication (Liu et al., 2013). Other oxysterols are also implicated in the antiviral response (Lembo et al., 2016). *In vitro* studies indicate 26-HC and 24S,25-epoxycholesterol (24S,25-EC) also have antiviral properties, but are less potent than 25-HC (Blanc et al., 2013; Cagno et al., 2017).

### 25-HC, Severe Acute Respiratory Syndrome Coronavirus-2 and Accessible Cholesterol

IFNs are induced by coronavirus infection (Park and Iwasaki, 2020), and IFN-stimulated genes are up-regulated in severe acute respiratory syndrome coronavirus (SARS-CoV) and SARS-CoV-2 infected cells (Lamers et al., 2020). One of these genes, *CH25H*, is found to be up-regulated in macrophages and lung epithelial cells found in bronchioalveolar lavage (BAL) fluid from COVID-19 patients (Wang et al., 2020). Besides infecting lung epithelia cells SARS-CoV-2 also infects intestinal epithelium cells (Lamers et al., 2020; Zang et al., 2020) and *CH25H* was found to be one of the IFN-stimulated genes in primary human enteroids (Zang et al., 2020). In a gene screen performed in HEK293-hACE2 cells (i.e., HEK-293 cells expressing the human ACE2 receptor, the receptor of SARS-CoV and SARS-CoV-2) *CH25H* supressed both SARS-CoV and SARS-CoV-2 pseudo-virus replication (see below; Zang et al., 2020). The product of CH25H enzymatic activity, 25-HC, has been shown to be elevated in some patients suffering from SARS-CoV-2 infection (Marcello et al., 2020; Zu et al., 2020) and 25-HC has been shown to be antiviral against SARS-CoV-2 by blocking spike protein catalysed membrane fusion (Wang et al., 2020; Zang et al., 2020). Besides 25-HC, it should be noted that other oxysterols including 26-HC and 7-oxocholesterol (also called 7-ketocholesterol) have been linked to the antiviral response against SARS-CoV-2 (Marcello et al., 2020; Ghzaiel et al., 2021).

SARS-CoV-2 is an enveloped single stranded RNA virus. It binds to the ACE2 (angiotensin converting enzyme 2) receptor and subsequently infects cells by either a plasma membrane or endosome fusion pathway. The plasma membrane fusion pathway requires the presence of membrane bound TMPRSS2 (transmembrane protease serine 2) to cleave the spike protein for early fusion (Hoffmann et al., 2020). SARS-CoV can enter cells *via* the endosomal pathway using the endosomal cysteine proteases cathepsin to cleave the spike protein, however, the TMPRSS2 mediated pathway may be dominant for SARS-CoV-2 membrane fusion (Hoffmann et al., 2020).

The experimental use of pathogenic SARS-CoV-2 virus demands strict biosafety levels, hence, the use of replication-restricted pseudo-viruses bearing viral coat proteins represent a safe alternative. Vesicular stomatitis virus (VSV), like SARS-CoV-2, is an enveloped virus but only causes mild flu-like symptoms. The VSV envelope G-protein can be replaced by a reporter gene bearing SARS-CoV-2 spike protein, giving, e.g., a VSV-eGFP-SARS-CoV-2 pseudo-virus, appropriate for studying viral entry mechanisms. VSV-SARS-CoV-2 pseudo-viruses have been extensively exploited in two recent studies on the mechanism of 25-HC antiviral activity against SARS-CoV-2 (Wang et al., 2020; Zang et al., 2020).

**Figure 12:**
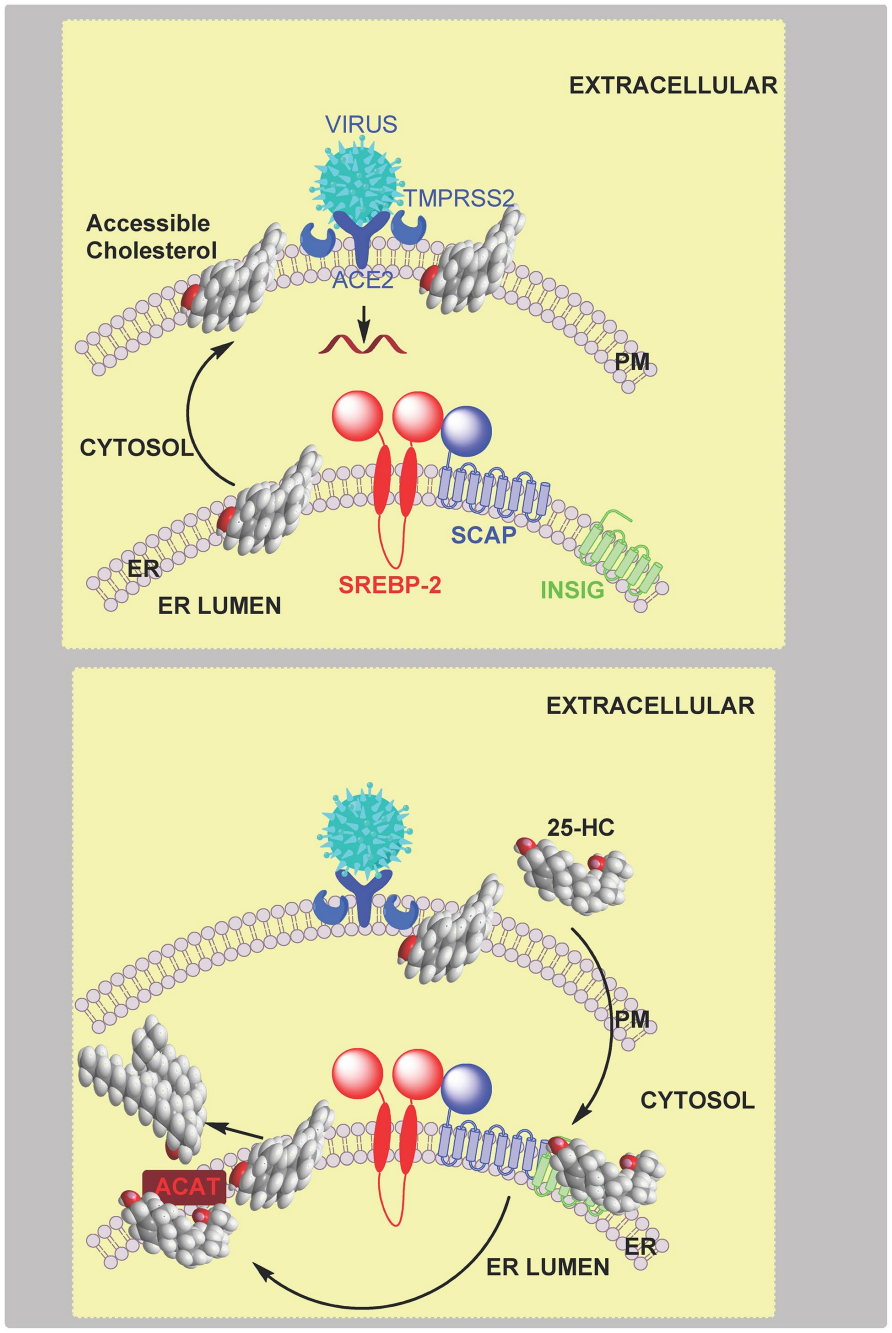
Model of coronavirus entry *via* the early pathway. **Upper panel**, virus binds to the cell surface ACE2 receptor. The membrane-bound TMPRSS2 enzyme triggers the early fusion pathway by proteolytic cleavage of the spike protein to induce the fusion-competent state of the protein. Membrane fusion proceeds and results in release of viral RNA into the cytoplasm. Membrane fusion is dependent on sufficient accessible cholesterol in the plasma membrane. **Lower panel**, 25-HC inhibits membrane-fusion and replication. This is achieved by reducing accessible cholesterol in the plasma membrane. Data from Wang et al. (2020) indicate that this is achieved by activating the endoplasmic reticulum enzyme ACAT/SOAT which esterifies cholesterol. An alternative explanation is 25-HC binding to INSIG and reducing cholesterol biosynthesis and up-take.

Focusing on the TMPRSS2 mediated early fusion pathway and human lung epithelial cells, 25-HC was found to inhibit SARS-CoV-2 pseudo-virus at a half-maximal inhibitory concentration (IC_50_) of 550nM (220ng/ml; Wang et al., 2020). Binding of the pseudo-virus to the host cell was not affected, but in a cell model of membrane fusion, 25-HC was shown to block plasma membrane fusion. Based on this data and studies demonstrating the involvement of 25-HC in the reduction of plasma membrane accessible cholesterol (Abrams et al., 2020), it was postulated that 25-HC blocks coronavirus spike-protein mediated membrane fusion by mobilising accessible cholesterol away from the plasma membrane (Wang et al., 2020). This mechanism may also be responsible for the antiviral activity of 25-HC against SARS-CoV and Middle East respiratory syndrome coronavirus (MERS-CoV), in addition to SARS-CoV-2 in lung epithelial cells where the plasma membrane fusion pathway is dominant. In experiments using Calu-3 cells, a lung epithelial cell line, where viral entry is *via* TMPRSS2, and using fluorescence-labelled ALOD4 as an indicator of accessible cholesterol, 25-HC was found to deplete accessible cholesterol in a dose dependent manner. Supplementation with cholesterol in complex with cyclodextrin, restored ALOD4 binding (Wang et al., 2020). Importantly, supplementing cholesterol also restored SARS-CoV-2 pseudo-virus entry in 25-HC treated cells, supporting the idea that 25-HC mobilises plasma membrane cholesterol to inhibit SARS-CoV-2 virus – plasma membrane fusion (Wang et al., 2020). Addition of cholesterol in complex with cyclodextrin also reverses inhibitory effects of 25-HC on Calu-3 cells challenged with SARS-CoV or MERS-CoV pseudo-virus (Wang et al., 2020). The explanation for the reduction of accessible cholesterol in response to 25-HC, may be a explained by (i) activation of LXRs and cellular cholesterol export; (ii) inhibition of SREBP-2 processing leading to a reduction in cholesterol biosynthesis and uptake *via* the LDL-receptor; (iii) stimulation of ubiquitination and proteolysis of HMG-CoA-reductase; and (iv) activation of ACAT/SOAT resulting in enhanced cholesterol ester formation (Figure 12). In Calu-3 cells, the depletion of plasma membrane accessible cholesterol resulting from 25-HC treatment is likely to be through activation of ACAT/SOAT, at least in lipid depleted medium, as the ACAT inhibitor Sandoz 58–035, reversed the reduction in accessible cholesterol and rescued SARS-CoV-2 pseudo-virus entry. ACAT/SOAT knockdown by shRNA also enhanced pseudo-virus entry in 25-HC treated cells (Wang et al., 2020). A caveat to these results is that the experiments were performed in lipid depleted medium, so any reduction in LDL-cholesterol uptake as a consequence of 25-HC inactivation of the SREBP-2 pathway leading to down-regulation of the LDL-receptor expression was not considered.

### 25-HC, SARS-CoV-2, and Endocytosis

**Figure 13:**
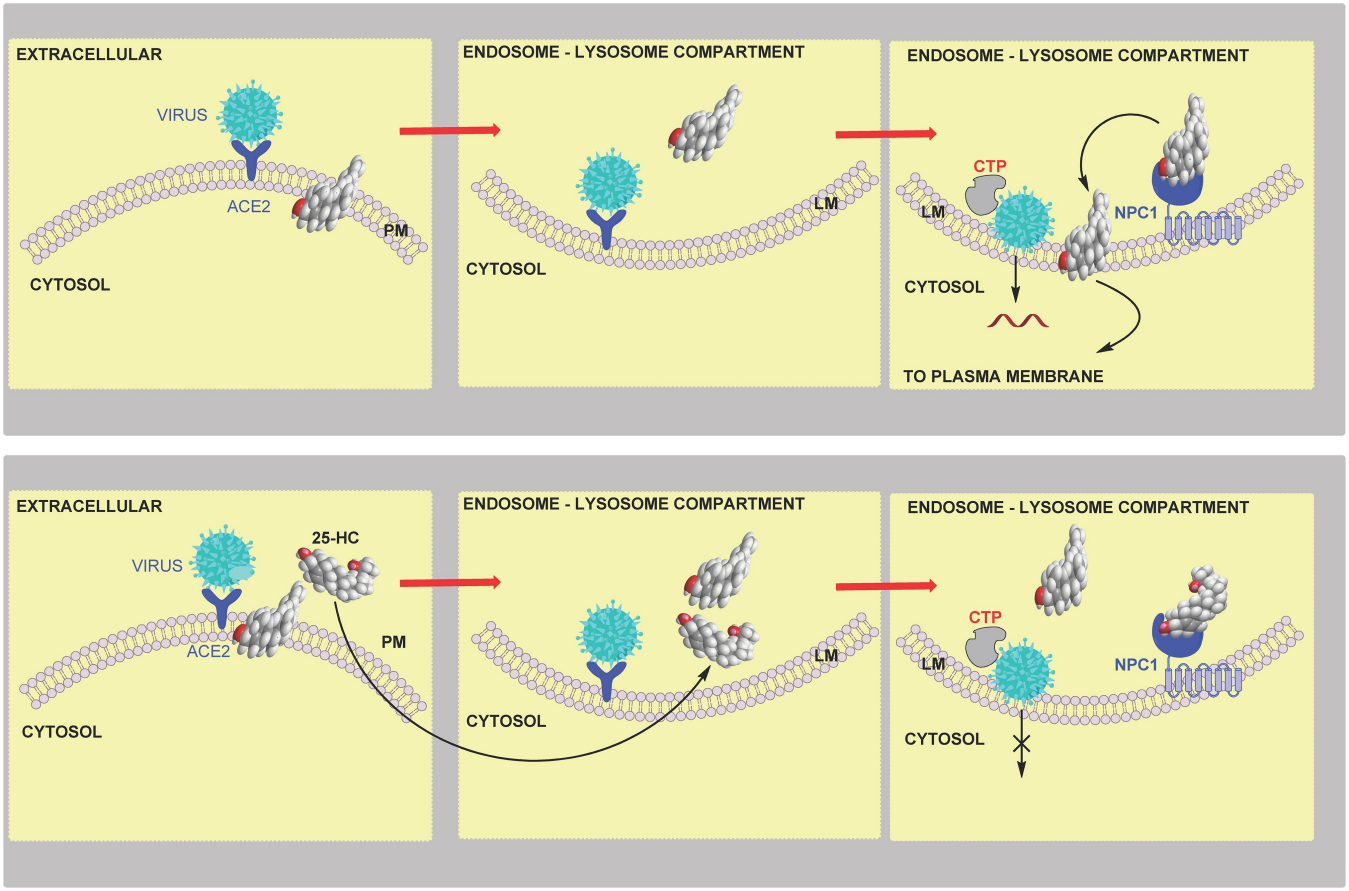
Model of coronavirus entry *via* the late endocytosis pathway. **Upper panel**, in the absence of TMPRSS2, virus is endocytosed and within the endosome-lysosome compartment low pH activates cathepsin (CTP) mediated cleavage of the spike protein triggering membrane fusion and release of viral RNA. **Lower panel**, 25-HC inhibits viral replication by binding to NPC1 and restricting cholesterol transport to the lysosome membrane and export, ultimately preventing membrane fusion and release of viral RNA.

An alternative route for coronavirus entry into cells is *via* endocytosis which also requires membrane fusion for viral RNA release (Hoffmann et al., 2020; Tang et al., 2020). 25-HC will inhibit viral replication by blocking the required membrane fusion event (Zang et al., 2020). *In vitro* cell fusion assays confirmed *CH25H* expression blocked membrane fusion, a result phenocopied by 25-HC. Interestingly, fluorescently-labelled 25-HC (C4 TopFluor-25HC), which was found to have an almost identical antiviral activity towards the SARS-CoV-2 pseudo-virus as 25-HC, was found to localise to late endosomes-lysosomes in HEK 293 cells. In addition, 25-HC treatment of these cells led to an accumulation of unesterified cholesterol (Zang et al., 2020). As discussed earlier, NPC1 protein transports unesterified cholesterol to the membrane of the lysosomal compartment, and will also bind 25-HC (Kwon et al., 2009). This data along with evidence that treatment with itraconazole (ICZ) and U18666A, two inhibitors of NPC1, which lead to reduced SARS-CoV-2 pseudo-virus infection, prompted the suggestion that cholesterol accumulation in the late-endosome-lysosome compartment may explain the antiviral activity of 25-HC (Figure 13). Importantly, the antiviral activity of 25-HC and ICZ are diminished in serum free medium, indicating that their antiviral activity is dependent on the accumulation of cholesterol in the endosomal-lysosomal compartment (Zang et al., 2020). A caveat to this interpretation of the data is that free 25-HC does not traverse the lysosome, at least not when it inhibits SREBP processing (Kwon et al., 2009). However, fluorescently-labelled 25-HC (C4 TopFluor-25HC) is a version of 25-HC esterified at C-25 through a linker to a fluorescent group, and may behave like other sterol esters and be taken-up by receptor mediated endocytosis at the LDL-receptor as part of LDL and enter the lysosome *via* this pathway while free 25-HC could proceed directly to the lysosome if not targeted to the endoplasmic reticulum. In plasma the majority of 25-HC is esterified (Dzeletovic et al., 1995), so if the circulation is the source of antiviral 25-HC then uptake by the LDL-receptor may provide a direct route to the lysosome. Furthermore, both ICZ and U18666A are non-specific inhibitors of NPC1, ICZ also inhibits CYP51, an enzyme in the cholesterol biosynthesis pathway and is an antagonist to Smoothened (SMO) a component of the hedgehog (Hh) signalling pathway (see section Oxysterols, Accessible Cholesterol, and Hedgehog Signalling; Kim et al., 2010). U18666A will also inhibit Aster proteins, important in the transport of accessible cholesterol from the plasma membrane to the endoplasmic reticulum (Xiao et al., 2021). However, experiments with U18666A were performed with epithelia cells not expressing TMPRSS2, hence mobilising plasma membrane accessible cholesterol would not be required to prevent viral entry in these cells (Zang et al., 2020).

When considered in concert, the results from the two studies (Wang et al., 2020; Zang et al., 2020) lead to a model where SARS-CoV-2 viral infection promotes IFN secretion and the expression of the IFN-stimulated gene *CH25H*. This leads to paracrine or autocrine action of 25-HC leading to inhibition of SARS-CoV-2 spike-protein mediated membrane fusion and inhibition of viral replication. Inhibition of membrane fusion can be mediated by reducing accessible cholesterol at both the plasma membrane and endosome-lysosome membrane level.

## Oxysterols, Accessible Cholesterol, and Hedgehog Signalling

Oxysterols have numerous roles in biological systems, but the most well defined are those involved in cholesterol homeostasis (Goldstein et al., 2006; Radhakrishnan et al., 2007; Brown et al., 2021; Wang et al., 2021). However, oxysterols have roles beyond cholesterol *per se* and can act as ligands to G protein-coupled receptors (GPCR; Hannedouche et al., 2011; Liu et al., 2011). One GPCR that oxysterols and cholesterol activate is Smoothened (SMO), a critical receptor in the Hedgehog (Hh) signalling pathway (Abdel-Khalik et al., 2020; Radhakrishnan et al., 2020). Like in cholesterol regulation and in protection against microbial infection, oxysterols and accessible cholesterol are closely linked in the Hh signalling pathway.

### Oxysterols and the Hedgehog Signalling Pathway

**Figure 14:**
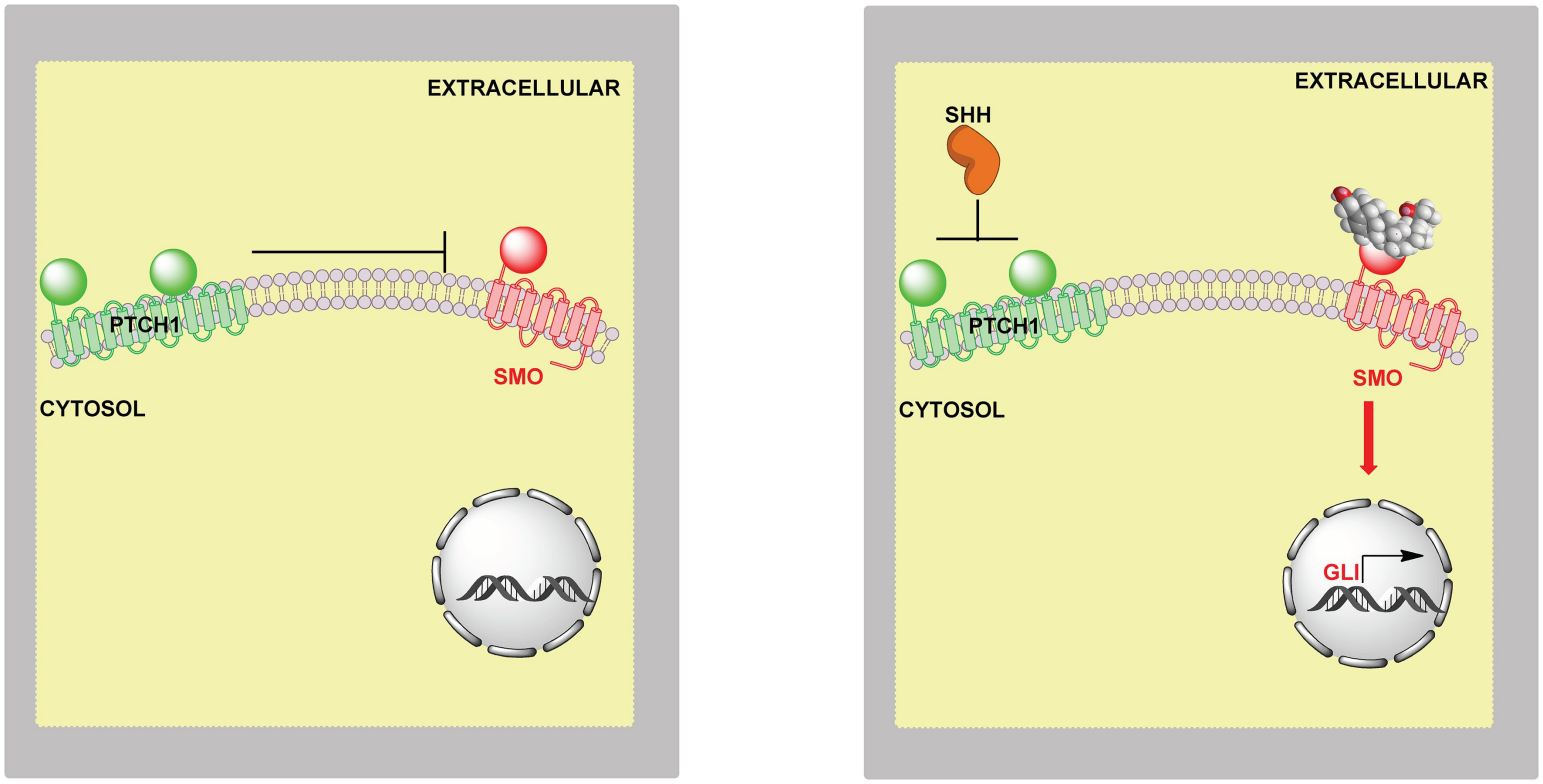
Model of the involvement of oxysterols in Hh signalling. **Left panel**, in the absence of extracellular SHH or oxysterols PTCH1 inhibits SMO and the Hh signal is not transmitted across the membrane. **Right panel**, SHH relives the inhibition by PTCH1 on SMO and oxysterols bind to SMO leading to activation of GLI target genes.

The Hh signalling pathway is an important coordinator of cell–cell communication required during development and regeneration (Radhakrishnan et al., 2020). Defects in the pathway can lead to disorders ranging from birth defects to cancers (Cooper et al., 2003; Raleigh et al., 2018). Hh ligands, e.g., Sonic Hedgehog (SHH) in vertebrates, initiate Hh signalling in a paracrine manner by binding to the extracellular side of Patched 1 (PTCH1), a 12-pass transmembrane protein. A second messenger then communicates the signal to the GPCR SMO, a 7-pass transmembrane protein, which transmits the signal across the membrane leading to the expression of GLI target genes (Kong et al., 2019; Radhakrishnan et al., 2020). In the absence of Hh ligand, PTCH1 inhibits SMO and prevents Hh signalling (Figure 14). The components for Hh signalling are found in primary cilia, antenna-like organelles which are continuous with the plasma membrane (Rohatgi et al., 2007). Side-chain oxysterols are potential second messengers in the Hh pathway, in that they activate Hh signalling in cultured cells, even in the absence of SHH, and induce the accumulation of SMO in primary cilia (Corcoran and Scott, 2006; Dwyer et al., 2007; Nachtergaele et al., 2012; Abdel-Khalik et al., 2020). Side-chain oxysterols bind to the extracellular cysteine-rich domain (CRD) of SMO (Myers et al., 2013; Nachtergaele et al., 2013; Nedelcu et al., 2013; Abdel-Khalik et al., 2020) and have also been found to be enriched in primary cilia (Raleigh et al., 2018). Cholesterol also binds to the CRD of SMO and has been considered as the second messenger between PTCH1 and SMO (Byrne et al., 2016; Luchetti et al., 2016). However, at first thought this is unlikely as cholesterol is so abundant in the plasma membrane. How could changes in cholesterol concentrations necessary for second messenger activity occur without disruption of normal membrane function, and could such changes avoid SREBP-2 regulation of cholesterol levels?

To investigate the nature of the second messengers involved in Hh signalling, a loss of function CRISPR screen was carried out in NIH/3T3 cultured cells targeting lipid-related genes (Kinnebrew et al., 2019). Cells were grown in lipoprotein depleted media and treated with U18666A, to block cholesterol up-take but enhance cholesterol biosynthesis (Kinnebrew et al., 2019). Enzymes of the cholesterol biosynthesis pathway were found to be positive regulators of Hh signalling, but surprisingly enzymes synthesising oxysterols from cholesterol were not found to be positive regulators of Hh signalling. This result needs to be considered in the context that the cultured cells were deprived of cholesterol, so machinery synthesizing oxysterols in these cells is not likely to be activated. An exception is 24S,25-epoxycholesterol (24S,25-EC) which is synthesised in parallel to cholesterol *via* a second epoxidation of squalene by squalene epoxidase (SQLE; Figure 6; Nelson et al., 1981). 24S,25-EC is a known activator of Hh signalling and binds to SMO (Raleigh et al., 2018; Qi et al., 2019). However, DHCR24 was found to be a positive regulator of Hh signalling (Kinnebrew et al., 2019), and as this is the one enzyme used to synthesize cholesterol but not 24S,25-EC (Figure 6; Nelson et al., 1981), this would suggest that cholesterol is a more likely second messenger between PTCH1 and SMO than 24S,25-EC, but not necessarily if the signalling is paracrine.

While enzymes of the cholesterol biosynthesis pathway were found to be positive regulators of Hh signalling, enzymes of sphingomyelin biosynthesis were found to be negative regulators (Kinnebrew et al., 2019).

### Accessible Cholesterol and the Hh Signalling Pathway

In the CRISPR screen, one of the top negative regulators was *Sptlc2* (serine palmitoyltransferase, long chain base subunit 2), which codes for the enzyme catalysing the first committed step in SM synthesis (Figure 5, lower panel). The fungal antibiotic myriocin will inhibit SPTLC2 and can be used to deplete SM in cells. Treatment of NIH/3T3 cells with myriocin was found to potentiate Hh signalling, confirming the involvement of SM in the Hh signalling pathway (Kinnebrew et al., 2019). Importantly, mutations in the CRD of SMO which abrogate cholesterol binding were found to reduce myriocin driven Hh signalling (Kinnebrew et al., 2019). This supports the concept of cholesterol through binding to SMO acting as the second messenger between PTCH1 and SMO.

The involvement of SM and cholesterol in the Hh signalling pathway suggests the involvement of accessible cholesterol. One model to explain the role SM and cholesterol in Hh signalling is that SM will sequester cholesterol making it inaccessible for Hh signalling thus reducing the availability of accessible cholesterol to act as the second messenger. Evidence to support this model comes from studies with ALOD4, the cholesterol binding domain of the bacterial toxin ALO, that can trap accessible cholesterol found on the outer leaflet of the plasma membrane (Infante and Radhakrishnan, 2017). ALOD4 was found to reduced Hh signalling in cultured cells, in contrast myriocin which blocks SM synthesis was found to enhance Hh signalling (Kinnebrew et al., 2019).

**Figure 15:**
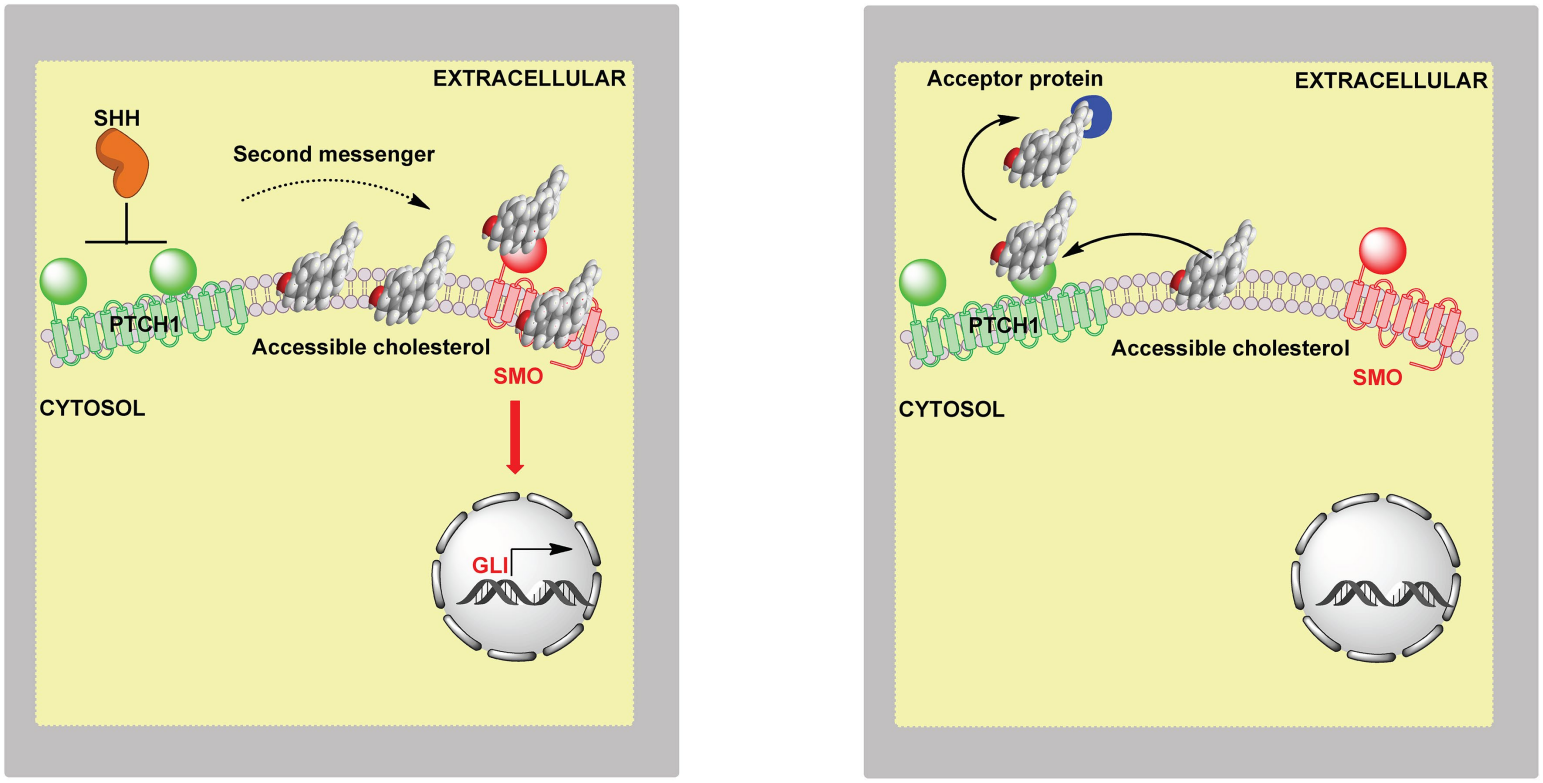
Cartoon representation of the involvement of accessible cholesterol in Hh signalling. **Left panel**, extracellular SHH binds to PTCH1 on the cilium membrane, SMO is activated and GLI transcription factors activate GLI target genes. When SHH binds to PTCH1 it inhibits the action of PTCH1, this may lead to accumulation of accessible cholesterol which acts as the second messenger between PTCH1 and SMO. Cholesterol can bind to the CRD and TMD of SMO, binding at both sites may be required for maximal activation. **Right panel**, the “pump leak” model proposes PTCH1 acts as a sterol pump removing sterols from the membrane in the vicinity of SMO, thereby preventing activation of SMO by sterols. Side-chain oxysterols cross membranes far faster than cholesterol and could potentially occupy one of the sterol binding sites in activated SMO. Oxysterols can activate SMO even in the absence of the SHH ligand.

If accessible cholesterol is the second messenger between PTCH1 and SMO how might its regulation be isolated from general plasma membrane cholesterol homeostasis controlled by SREBP-2? One explanation is that the machinery for Hh signalling is located in primary cilia (Rohatgi et al., 2007). Primary cilia have a distinct protein and lipid composition from the bulk plasma membrane to which they merge, including a different SM and accessible content from the rest of the membrane (Nachury and Mick, 2019). Using PFO* as a probe for accessible cholesterol, OlyA [Ostreolysin A, a non-lytic fungal toxin which selectively binds SM sequestered cholesterol (Endapally et al., 2019)] as a probe for SM in complex with cholesterol, and OlyA-E69A (a mutant of OlyA that binds free SM and cholesterol-complexed SM) as a probe for total SM, it was shown that the SM to cholesterol ratio was high in primary ciliary membranes and that myriocin treatment increased the amount of accessible cholesterol relative to the bulk plasma membrane (Kinnebrew et al., 2019). This led to the suggestion that SM in primary cilia is critical for keeping SMO inactive (Kinnebrew et al., 2019). To prevent continuous signalling between PTCH1 and SMO, how might a low level of accessible cholesterol be maintained in primary cilia and how might this be independent from the bulk plasma membrane? One explanation is the “Pump-Leak” model where PTCH1 keeps accessible cholesterol below a threshold for SMO activation by transporting accessible cholesterol out of cilia and on to intra- or extracellular acceptors (Kinnebrew et al., 2019; Radhakrishnan et al., 2020). PTCH1 is a sterol transporter and negatively regulates sterol access to the CRD of SMO (Xiao et al., 2017). When PTCH1 is inactivated by the SHH ligand, accessible cholesterol leaks into the cilia and activates SMO (Figure 15, left panel). PTCH1 shows sequence similarity to the cholesterol transporter NPC1 which transports cholesterol out of the lysosome. Both have a sterol sensing domain. NPC1 is suggested to transport cholesterol through a protein tunnel leading to the outer leaflet of the lysosomal membrane (Qian et al., 2020), the soluble protein NPC2 having delivered cholesterol to the N-terminal domain of NPC1. PTCH1 could transport cholesterol in an opposite manner from the outer leaflet of the plasma membrane to a protein acceptor, or alternatively, receive cholesterol from SMO and transport it to the membrane (Radhakrishnan et al., 2020).

It is still not clear how accessible cholesterol gains access to SMO, from the inner or outer membrane leaflet. Besides the extracellular CRD (Byrne et al., 2016; Huang et al., 2016), cholesterol has also been shown to bind to the transmembrane domain (TMD) of SMO with access suggested to be from the inner membrane (Deshpande et al., 2019). Probably, PTCH1 inactivation leads to increased accessible cholesterol in both leaflets with cholesterol flip flopping between the two, and either route to SMO activation is possible (Radhakrishnan et al., 2020). In fact, sterol binding to the CRD and TMD may be required for full activation, alternatively one site may be constitutively bound to promote SMO stability while occupation of the second is regulated by PTCH1. A cryo-EM structure of SMO has been solved with 24S,25-EC in the TMD (Qi et al., 2019). Could this be the true regulator of Hh signalling while cholesterol in the CRD is required to promote stability or vice versa?

### Back to Oxysterols

Side-chain oxysterols bind to and activate SMO in cultured cells (Corcoran and Scott, 2006; Dwyer et al., 2007; Nachtergaele et al., 2012; Myers et al., 2013; Nedelcu et al., 2013; Raleigh et al., 2018; Abdel-Khalik et al., 2020). Could oxysterols and cholesterol work in concert with one molecule occupying the CRD pocket and the other the TMD and together promote maximum signalling? This is not an unreasonable concept as Smith-Lemli-Opitz syndrome (SLOS) which presents with dysmorphology consistent with defective Hh signalling is accompanied by reduced cholesterol biosynthesis and an unusual pattern of oxysterols. Some of the SLOS derived oxysterols which are modified in both the sterol side-chain and ring fit into the CRD binding pocket of SMO and activate Hh signalling, but are perhaps less efficient activators than simple side-chain oxysterols, resulting in reduced Hh signalling during development of the SLOS embryo compared to that experienced during normal development (Abdel-Khalik et al., 2020). In addition, there is good evidence that oxysterols oxidised in the ring and side-chain activate SMO in the context of medulloblastoma (Raleigh et al., 2018). With regard to the action of oxysterols and cholesterol in Hh signalling it should be noted that activation of LXR by the classic pharmacological ligands TO901317 and GW3965 has been found to inhibit Hh signalling (Kim et al., 2009). TO901317 was found to induce the expression of LXR target genes *Abca1* and *Abcg1* but inhibit the expression of SHH-induced target genes *Ptch1* and *Gli1* (Kim et al., 2009). It is not clear whether LXR activation by oxysterols will have a similar effect on Hh signalling, but if so this may proceed *via* ABCA1 or ABCG1-mediated transport of accessible cholesterol from cilia.

Side-chain oxysterols are often considered as a transport forms of cholesterol, in similar vein they could also be considered as an secondary form of accessible cholesterol in that they are not sequestered by SM (Endapally et al., 2019), they rapidly move through membranes (Lange et al., 1995) and could provide a paracrine form of signalling.

## Relationship Between Oxysterols and Accessible Cholesterol

In relation to binding to SMO and activating Hh signalling, side-chain oxysterols can be considered as a rapidly available secondary form of accessible cholesterol. They may be most important with respect to paracrine signalling as they are transferred across membranes orders of magnitude faster than non-esterified cholesterol (Lange et al., 1995; Meaney et al., 2002). This concept of oxysterols as a rapidly available form of signalling accessible cholesterol can be extended further to the regulation of cellular cholesterol by the SREBP-pathway. Side-chain oxysterols will behave like plasma membrane accessible cholesterol and inhibit SREBP-2 processing, the difference being that side-chain oxysterols can move across membranes much quicker than free cholesterol and are thus more likely to have an immediate effect, important for fine tuning of the pathway (Gill et al., 2008).

### Importance of Measuring Free and Esterified Oxysterols

During *in vitro* studies oxysterols are added to cells in a non-esterified form, this can be regarded as a mimic of autocrine, paracrine or hormonal signalling. To assess the physiological relevance of the oxysterol, comparisons are made between concentrations required to have a biological effect and those present in a biological tissue or fluid (e.g., plasma). However, confusion can arise if the concentration of oxysterol in the biological material is not clearly defined as being non-esterified or total (esterified plus non-esterified), as it is the non-esterified oxysterol that rapidly crosses membranes. If cells are grown in medium that is replete in lipoproteins, the LDL-receptor will not be expressed, and esterified oxysterols cannot enter cells *via* this route. On the other hand, if cells are grown in lipid depleted medium, then the LDL-receptor will be expressed and oxysterols can be taken up *via* receptor mediated endocytosis along with cholesterol esters. However, release from the lysosome will require prior ester hydrolysis.

## Conclusion

In this review we have considered the growing importance of the concept of accessible cholesterol in relation to cholesterol homeostasis. The concept can be used to explain how oxysterols are important in defence against microbial pathogens and also how cholesterol itself can act as a second messenger during development. We have paid particular attention to how accessible cholesterol is transported from the plasma membrane to the endoplasmic reticulum. While this system has been well studied, transport of oxysterols in cells has been less extensively studied. In this regard oxysterol binding proteins (OSBP) and the family of OSBP-related (ORP) or OSBP-like (OSBPL), which as their name suggest bind oxysterols, are likely to be important (Olkkonen and Hynynen, 2009).

With respect to oxysterols and the immune system we have discussed how macrophages in response to activation of pattern recognition receptors by pathogens secrete 25-HC, an action also performed by dendritic cells (Bauman et al., 2009; Diczfalusy et al., 2009; McDonald and Russell, 2010; Park and Scott, 2010; Blanc et al., 2013; Liu et al., 2013; Cyster et al., 2014; Dang et al., 2017). 25-HC can have antimicrobial activity protecting endothelial and epithelial tissue and macrophages and neutrophiles from infection (Abrams et al., 2020; Wang et al., 2020; Zang et al., 2020; Zhou et al., 2020). 25-HC and also 26-HC may be natural antivirals against SARS-CoV-2 (Marcello et al., 2020; Wang et al., 2020; Zang et al., 2020), and we await detailed studies of the oxysterol profiles of infected lung tissue. This will not be easy as lung tissue is highly vascular presenting a problem in distinguishing oxysterols derived from tissue from those present in the circulation. The literature on oxysterol patterns in lung tissue is rather sparse; however, the oxysterol profile of mouse lung has recently been reported following LPS treatment (Bottemanne et al., 2021), while the oxysterol patterns in bronchoalveolar lavage fluid from patients with mild asthma 48h after acute allergen challenge have been measured (Shen et al., 2017). In human the most well-established oxysterol derivative generated in lung is 3β-hydroxycholestenoic acid (Babiker et al., 1999). Interestingly, 3β-hydroxycholestenoic acid is abundant in the circulation, is an LXR agonist (Song and Liao, 2000), and has been shown to be a potent γ-secretase modulator and could protect against Alzheimer’s disease (Jung et al., 2015). The involvement of oxysterols in brain has been discussed elsewhere, but the concept of accessible cholesterol in neuronal development and degeneration has yet to be considered (Wang et al., 2021).

In summary, in this review we have attempted to draw together the concept of signalling *via* accessible cholesterol with that of side-chain oxysterols as paracrine and rapid signalling forms of accessible cholesterol, resurrecting to some extend the oxysterol hypothesis of Kandutsch, Chen and Heiniger (Kandutsch et al., 1978). We have also tried emphasising the protective effects of oxysterols against pathogens and the involvement of oxysterols in modulating accessible cholesterol to provide protection.

## Author Contributions

All authors listed have made a substantial, direct and intellectual contribution to the work, and approved it for publication.

## Funding

This work was supported by the UKRI Biotechnology and Biological Sciences Research Council (BBSRC; grant numbers BB/I001735/1 and BB/N015932/1 to WG and BB/L001942/1 to YW), and the Welsh Government and the European Union through European Structural Funds.

## Conflict of Interest

The authors declare that the research was conducted in the absence of any commercial or financial relationships that could be construed as a potential conflict of interest.

## Publisher’s Note

All claims expressed in this article are solely those of the authors and do not necessarily represent those of their affiliated organizations, or those of the publisher, the editors and the reviewers. Any product that may be evaluated in this article, or claim that may be made by its manufacturer, is not guaranteed or endorsed by the publisher.
